# The increasing role of drought as an inciting factor of bark beetle outbreaks can cause large-scale transformation of Central European forests

**DOI:** 10.1007/s10980-025-02125-w

**Published:** 2025-05-23

**Authors:** Agnish Kumar Das, Marco Baldo, Laura Dobor, Rupert Seidl, Werner Rammer, Roman Modlinger, Prosper Washaya, Katarína Merganičová, Tomáš Hlásny

**Affiliations:** 1 Faculty of Forestry and Wood Sciences, Czech University of Life Sciences in Prague, Kamýcka 129, 165 00, Prague 6, Suchdol, Czech Republic; 2 Ecosystem Dynamics and Forest Management, Technical University of Munich, TUM School of Life Sciences, Hans-Carl-Von-Carlowitz-Platz 2, 85354 Freising, Germany

**Keywords:** Forest disturbance, Climate change, iLand model, *Ips typographus*, Norway spruce

## Abstract

**Context:**

Historically, large-scale outbreaks of the European spruce bark beetle were initiated mainly by windthrows. However, after 2018, a severe drought triggered the hitherto largest bark beetle outbreak observed in Europe, signalling a major shift in the disturbance regime.

**Objectives:**

Develop and test an approach that allows simulating this novel disturbance dynamics and evaluate landscape-scale compound impacts of wind- and drought-initiated outbreaks throughout the twenty-first century.

**Methods:**

We incorporated drought-initiated outbreaks into the forest landscape simulation model iLand, using critical values of vapour pressure deficit as the outbreak trigger. Forest management records and remote sensing-based disturbance maps were used to derive model parameters and evaluate simulated dynamics in a Central European forest landscape (41,000 hectares). The period 1961–2021 was used for model evaluation, and the years until 2100 for scenario analysis.

**Results:**

**I**ncorporating drought as outbreak trigger led to a notable decoupling of wind and bark beetle disturbances, which have historically formed a typical disturbance cascade in European forests. While forest growing stock and species composition were resilient to a wind-dominated disturbance regime, this resilience diminished under the compounded impact of wind- and drought-triggered disturbances. The new disturbance regime caused a persistent decline in Norway spruce and resulted in an overall decrease in landscape-level growing stock.

**Conclusions:**

Our findings underscore the urgent need for new approaches to evaluate increasingly complex disturbance dynamics and suggest that the future impacts of bark beetles on forest landscapes may be greater than previously anticipated.

**Supplementary Information:**

The online version contains supplementary material available at 10.1007/s10980-025-02125-w.

## Introduction

After 2018, Europe has experienced an unprecedented wave of tree mortality that significantly departed from the historical dynamics in many regions (Senf and Seidl 2021). This disturbance pulse was spatially aligned with soil moisture and vapour pressure deficit anomalies, implying an increasing role of drought in disturbance regimes (Senf et al. 2020). Drought legacy effects observed in the following years suggest that physiological recovery processes were impaired, and trees were prone to secondary impacts from pests and pathogens (Schuldt et al. 2020).

The most severe and widespread disturbances in European forests have been caused by the European spruce bark beetle (*Ips typographus* L.), which primarily affects Norway spruce (*Picea abies* (L.) H. Karst.), a species constituting approximately 25% of the total growing stock in Europe’s forests (Hlásny et al. 2021). *I. typographus* belongs to the subfamily Scolytinae (Coleoptera: Curculionidae) and is one of the few bark beetle species exhibiting eruptive population dynamics, capable of rapidly increasing in number and causing extensive tree mortality (Raffa et al. 2008). During outbreaks, enlarged beetle populations overwhelm tree defences through mass attacks, coordinated by chemical signalling that guides individuals to specific host trees (Raffa et al. 2016). The beetle typically targets trees older than 60 years and with diameter exceeding 20 cm (Wermelinger 2004). Under outbreak conditions and high population densities, smaller and younger trees may also be colonized. Factors such as a high proportion of spruce, high stand density, advanced stand age, and the presence of forest edges are among the key conditions that predispose bark beetle infestation on the stand level (Netherer and Nopp-Mayr 2005).

*I. typographus* possesses several traits regulated by temperature and photoperiod, such as spring swarming, generation timing, and voltinism, which allow it to adapt its life cycle across a broad geographic range (Baier et al. 2007; Bentz et al. 2019). In many parts of Europe, where the annual heat sum is sufficient, *I. typographus* can produce more than one generation per year (Jönsson et al. 2009). While warmer climate increases the number of annually completed bark beetle generations and extends the beetles` flight period (Jakoby et al. 2019), heat and drought spells compromise the defence mechanisms of host trees (Huang et al. 2020), Netherer et al. 2021. Specifically, spruce cannot tolerate a drop in plant water potential below a certain limit (Schumann et al. 2024), at which point it closes stomata to prevent further water loss. This stomatal closure, typical of spruce’s isohydric strategy, helps protect the tree against hydraulic failure, but also limits photosynthesis and thus the production of defence compounds against bark beetles (Hartmann et al. 2018; Huang et al. 2020). Hot and dry conditions can thus boost population growth of *I. typographus*.

Historically, large outbreaks of *I. typographus* were mainly initiated by windthrows, which generated pulses of breeding material that boosted beetle population growth (Wermelinger 2004). Upon successful colonization of windfelled trees, the enlarged beetle populations typically expanded into the surrounding forests and caused tree mortality that often exceeded the impact of the initial windthrow (Mezei et al. 2017). The outbreaks triggered by windthrows typically declined after several years due to the exhaustion of local resources and other mechanisms, such as decreasing fitness of individual beetles, intra-specific competition, increasing levels of antagonists, defence priming of host trees and changes in symbiont communities (Biedermann et al. 2019).

The recent emergence of drought-initiated outbreaks occurring in the absence of windthrows represents a distinct change away from the historical disturbance regime. Such drought-induced outbreaks have occurred across a range of environments and forest types in recent years, including lowland and montane forests of Czechia, Germany, Austria, France, Italy, and Sweden (Hlásny et al. 2021; Kärvemo et al. 2023; Washaya et al. 2024). They have severe ecological and socio-economic consequences, including accelerated species turnover, reduced forest carbon stocks and increased rate of carbon release to the atmosphere, compromised cultural ecosystem values and increased timber price fluctuations (Buras et al. 2020; Hlásny et al. 2021; Asada et al. 2023). While climate projections do not indicate any clear trend in windspeed (Seneviratne et al. 2021), suggesting a relatively stable frequency of outbreaks induced by windthrows, a strong trend in climate aridification throughout the distribution of Norway spruce (Vicente-Serrano et al. 2013) suggests an increasing frequency of drought-induced outbreaks. The amplifying effect of climate change on outbreak size and severity together with the increased incidence of triggering events can thus lead to a substantial increase in forest disturbance by bark beetles. However, the possible future trajectories and their implications for the provisioning of ecosystem services remain unclear.

Dynamic vegetation models are important tools for understanding ecosystem dynamics under climate change (Blanco and Lo 2023; Rammer et al. 2024). However, models are challenged by the emergence of novel process interactions and feedbacks, which require a continuous evaluation of model structure. In the context of *I. typographus* dynamics, models exist for simulating the interaction between windthrows and outbreaks, for example, PICUS, iLand, and LandClim (Temperli et al. 2013; Maroschek et al. 2015; Rammer et al. 2024). Water limitation typically serves as predisposing or amplifying factor in these models but is not considered as an inciting factor (sensu Manion 1981) of large-scale outbreaks. Given the experiences of years following 2018 and the expected future increase in drought frequency and severity, this design element of current models could lead to a considerable underestimation of bark beetle dynamics under climate change. Considering drought as predisposing, amplifying, and inciting factor of outbreaks explicitly could thus significantly improve simulations of climate change-induced shifts in disturbance regimes.

Here, our objectives were to (i) develop an approach that incorporates drought-induced bark beetle outbreaks into the forest landscape and disturbance model iLand (Seidl et al. 2012; Rammer et al. 2024); (ii) test whether this approach is able to capture important characteristics of the recent bark beetle outbreak in Central Europe; (iii) analyze simulations incorporating the compound effects of wind- and drought-initiated outbreaks throughout the twenty-first century considering scenarios of climate change; and (iv) assess their impact on forest species composition and growing stock.

## Methods

### Simulation model

iLand is a process-based forest landscape model that simulates forest dynamics at the level of individual trees for landscapes spanning several thousands of hectares (Seidl et al. 2012; Rammer et al. 2024). The model accounts for continuous processes such as tree growth, mortality, and regeneration, and discontinuous processes such as natural disturbances and forest management. iLand operates in a multiscale hierarchical framework, with large-scale processes forming constraints for processes at finer scales, and the dynamics at fine scales feeding into processes at higher scales.

iLand dynamically simulates the regeneration, growth and mortality of individual trees, as influenced by climate, soil, initial state of the vegetation and disturbance. The spatial resolution of forest dynamics simulations is 2 × 2 m, while element cycles and environmental constrains related to energy, water and nutrients are considered at the scale of 100 × 100 m. iLand is driven by daily climate data, i.e., minimum and maximum air temperature, precipitation, radiation, and vapour pressure deficit. Production physiology is modelled using a light-use efficiency approach, driven by environmental conditions and species traits (Landsberg and Waring 1997). Carbohydrate allocation in trees is calculated annually based on allometric ratios and is sensitive to a tree’s competitive status. Tree regeneration is modelled at an annual time step in 2 × 2 m^2^ grid cells. Seed dispersal, climate-dependent establishment, and seedling and sapling growth are driven by species-specific traits. The mortality probability of a tree is influenced by its carbon balance, size, and age.

Management is simulated using iLand’s Agent-Based Management Engine (Rammer and Seidl 2015), which employs so-called Stand Treatment Programs (STPs) to execute a series of silvicultural operations throughout the course of stand development. STPs include planting, thinning and harvesting of trees. The adaptive elements of STPs include modifying the timing of thinning based on the actual stand density, replanting disturbed sites depending on the state of natural regeneration, and resetting the sequence of operations if a stand experiences severe disturbance.

Wind disturbances are simulated based on maximum wind speed, wind direction, and storm duration (Seidl et al. 2014). Impacts are simulated with a dose–response model, accounting for the vertical wind profile and resulting turning moment (Gardiner et al. 2000), and for local sheltering by neighbouring trees as well as the size of upwind gaps. Wind disturbances are initiated in locations where vertical differences between the top heights of neighbouring grid cells exceed 10 m, i.e., at forest edges (Blennow and Sallnäs 2004). Critical wind speeds are calculated separately for stem breakage and uprooting based on Gardiner et al (2000). Wind impacts are simulated iteratively during an individual event, with forest structure being updated after each iteration. The model was tested, for example, in Sweden following the storm Gudrun (2005), displaying good correspondence of observed and predicted wind damage patterns (Seidl et al. 2014).

iLand includes an advanced submodule for simulating European spruce bark beetle (*Ips typographus* L.) dynamics. The module considers the phenology and development of beetles, their spatially explicit dispersal, host tree colonization and defence, and temperature-related overwintering success (Seidl and Rammer 2017). Beetle development is simulated using a phenology-based process model (Baier et al. 2007), accounting for life-stage specific thermal requirements for beetle development and determining the number of generations and sister broods the insect can complete per year. Host trees are Norway spruce trees with a diameter at breast height of > 15 cm. Concerning dispersal, the model tracks beetle cohorts defined as the minimum number of beetles needed to colonize a tree. Every brood tree disperses a number of beetle cohorts determined by the reproductive rate of the beetles, estimated to range between 4 and 24 (Wermelinger and Seifert 1999). Dispersal consists of two stages: a passive flight simulated with a symmetrical dispersal kernel and an active flight where beetles look for suitable host trees within a 30 × 30 m search window (Kautz et al. 2014). Host colonization success depends on the defense capacity of the host tree that is approximated by non-structural carbohydrate reserves (Huang et al. 2020). While bark beetle population size fluctuates depending on meteorological conditions, outbreaks are mainly triggered by windthrows, which provide a surplus of breeding material (Wermelinger 2004). Removal of windfelled trees can thus exert a strong dampening effect on bark beetle outbreaks (Dobor et al. 2020a; Augustynczik et al. 2021). Model simulations were previously tested against independent data by Sommerfeld et al. (2021), who found good correspondence of simulated levels of infestation and spatial infestation patterns. The model code and executable as well as an extensive online documentation are available at https://iland-model.org/.

### Implementation and testing of drought-initiated outbreaks

In the absence of windthrow, bark beetle infestations are initiated in iLand based on a parameter determining the probability of a forest patch to be infested, represented as the annual outbreak probability per hectare. This initial probability (IP) is a user parameter with a default value of 0.000685, which has been estimated based on an empirically determined disturbance rotation period of 365 years and a mean disturbance size of 4 ha (Thom et al. 2013; Thom and Seidl 2016). The IP is modified by a climate-sensitive modifier that represents the deviation of summer precipitation in the actual year from the historical average conditions for which the IP has been specified (Seidl et al. 2016). While these fluctuations can mimic the variation in the size of endemic bark beetle populations (i.e., in a non-outbreak phase), they are unable to initiate large-scale outbreaks.

**Fig. 1:**
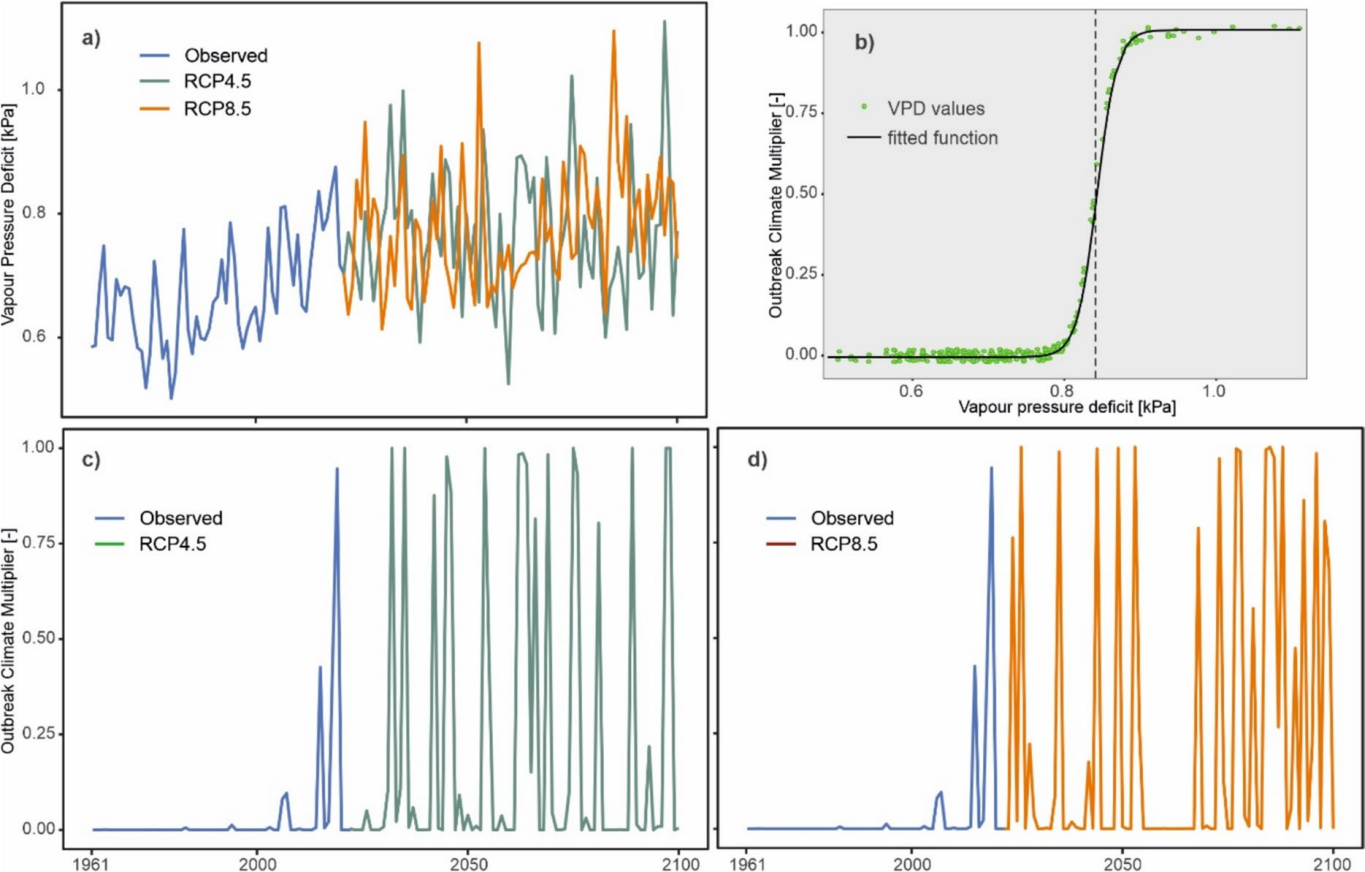
A scheme for calculating the Outbreak Climate Multiplier (OCM) used to initiate bark beetle outbreaks in iLand. **a** Time series of mean June-July vapour pressure deficit (VPDjj) for the period 1961–2100, consisting of observed data until 2021 and climate projections produced by the model EC-EARTH_RACMO22E driven by greenhouse gas concentration scenarios RCP4.5 and RCP8.5 afterwards. **b** A sigmoidal function transforming VPDjj into the OCM. The vertical dashed line represents the critical VPDjj value initiating the outbreaks, here set to 0.84 kPa. **c**, **d** Resulting OCM time series indicating conditions with elevated potential for the outbreaks

We build on the existing iLand bark beetle module – as described above – for incorporating drought as an inciting factor of bark beetle outbreaks. Since research on drought-induced outbreaks of spruce bark beetle remains scarce (but see Hlásny et al. 2021; Kärvemo et al. 2023; Washaya et al. 2024), we used a semi-empirical model formulation, combining fundamental ecological understanding with limited observational data (Jorgensen and Bendoricchio 2001). Specifically, we introduced a mechanism for drought-initiated outbreaks using Vapour Pressure Deficit (VPD) as an indicator for drought, as VPD is a strong driver of tree mortality (Park Williams et al. 2013; Hartmann et al. 2018). Moreover, VPD is readily available within iLand, as it affects stomatal conductance and primary production in the model (Rammer et al. 2024). We introduced a new function to the module, the *OutbreakClimateMultiplier* (OCM), which increases IP during heavy droughts. Specifically, the function transforms VPD into an OCM value, which is then used to scale IP multiplicatively (Fig. 1, Eq. 1). Rather than using mean annual VPD, we used the biologically more relevant June-July mean VPD (*VPDjj*), roughly corresponding to the time of year when the second bark beetle generation emerges in Central Europe (Holuša et al. 2012), i.e., during a period when increased climatic stress to trees can critically boost beetles` colonization success and population growth. As the interannual fluctuations in *VPDjj* led to unrealistic model behaviour and to consider the effect of multi-year droughts we applied a weighted *VPDjj*, using 70% from the current year and 30% from the previous year (VPDjj = 0.7*VPDjj_year i_ + 0.3*VPDjj_year i-1_). This aligns with documented effects of multiyear droughts on tree defence, although critical drought duration thresholds remain unclear (Peltier et al. 2023; Netherer et al. 2024). The modification of IP by the OCM is applied in a spatially explicit manner at the level of iLand resource units (i.e., 1-ha grid cells seamlessly defining environmental conditions across the study landscape). Once an outbreak is initiated, its further development is governed by the processes described earlier. Hence, once triggered, drought-induced outbreaks in the model follow a development pattern similar to wind-induced outbreaks.

The transformation of VPDjj into the OCM is conducted by means of a sigmoidal function:

1 $$OCM = k\frac{1}{{1 + e^{ - a(x - b)} }}$$

where OCM is the *Outbreak Climate Multiplier*, *b* is a critical VPD value defining the function`s inflection point, *a* is the slope of the function (Fig. 1), *k* is a multiplier to scale OCM to result in a maximum outbreak probability that corresponds to that of windfelled trees, and *x* is *VPDjj*.

To address the uncertainty of parameter definitions, we represented each parameter in Eq. 1 as a distribution function:

$$k \sim N(\mu_{k} ,\;\sigma_{k}^{2} ),\;a \sim N(\mu_{a} ,\;\sigma_{a}^{2} ),\;b \sim N(\mu b,\;\sigma_{b}^{2} )$$

where N(μ_k_, σ_k_^2^) represents a normal distribution of parameter *k,* with μ_k_ as the mean and σ_k_^2^ as the variance. The same notation applies to the parameters *a* and *b*. In each simulation, a parameter value is randomly drawn from the corresponding distribution and translated into the OCM. This procedure is applied individually to each 1-hectare resource unit, for which environmental constraints, including climate, are defined.

**Fig. 2:**
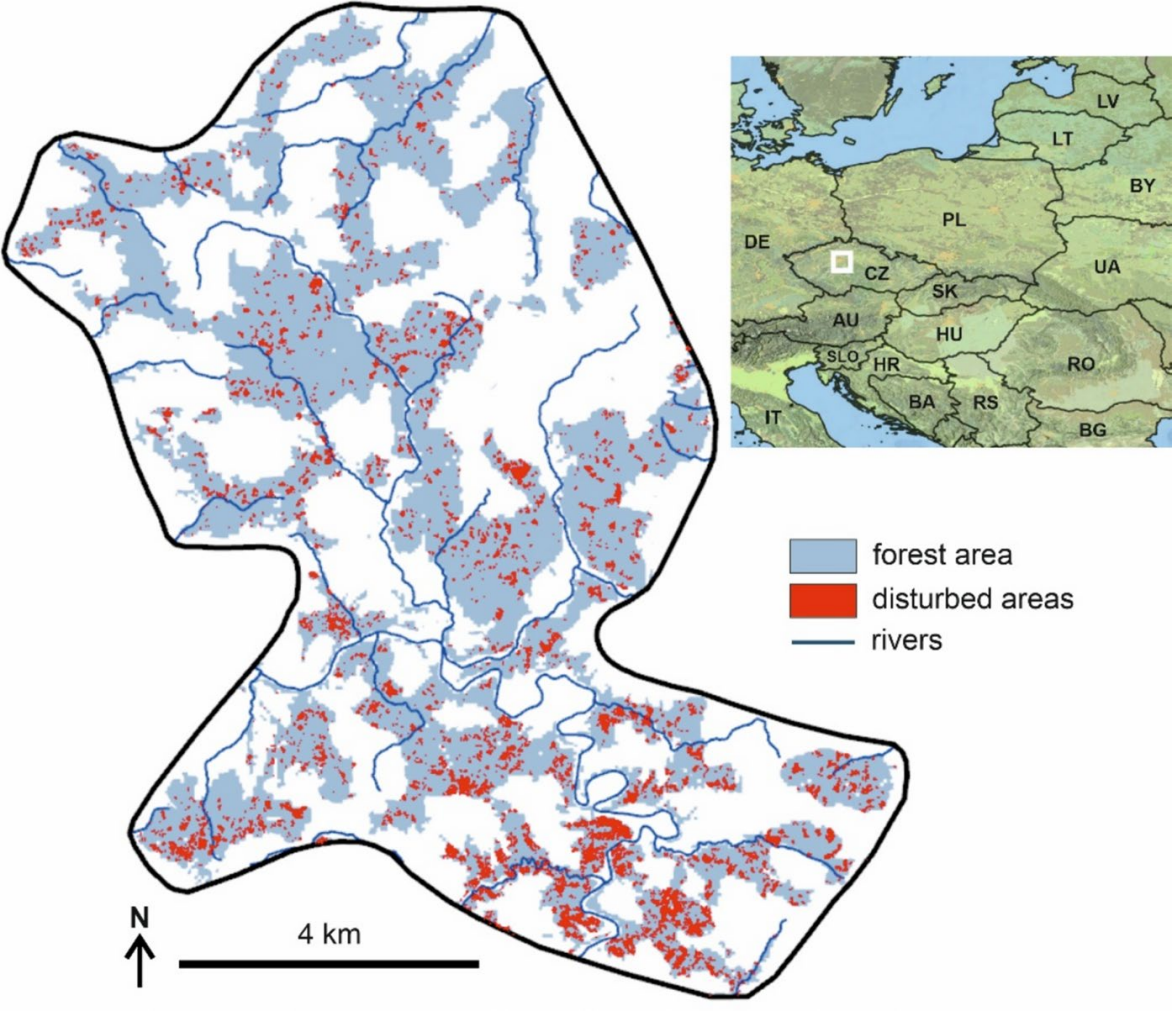
The position of the study landscape in Europe (a white square), forest cover of the landscape and the extent of bark beetle disturbance between 2018 and 2021 identified based on classification of satellite data

**Fig. 3:**
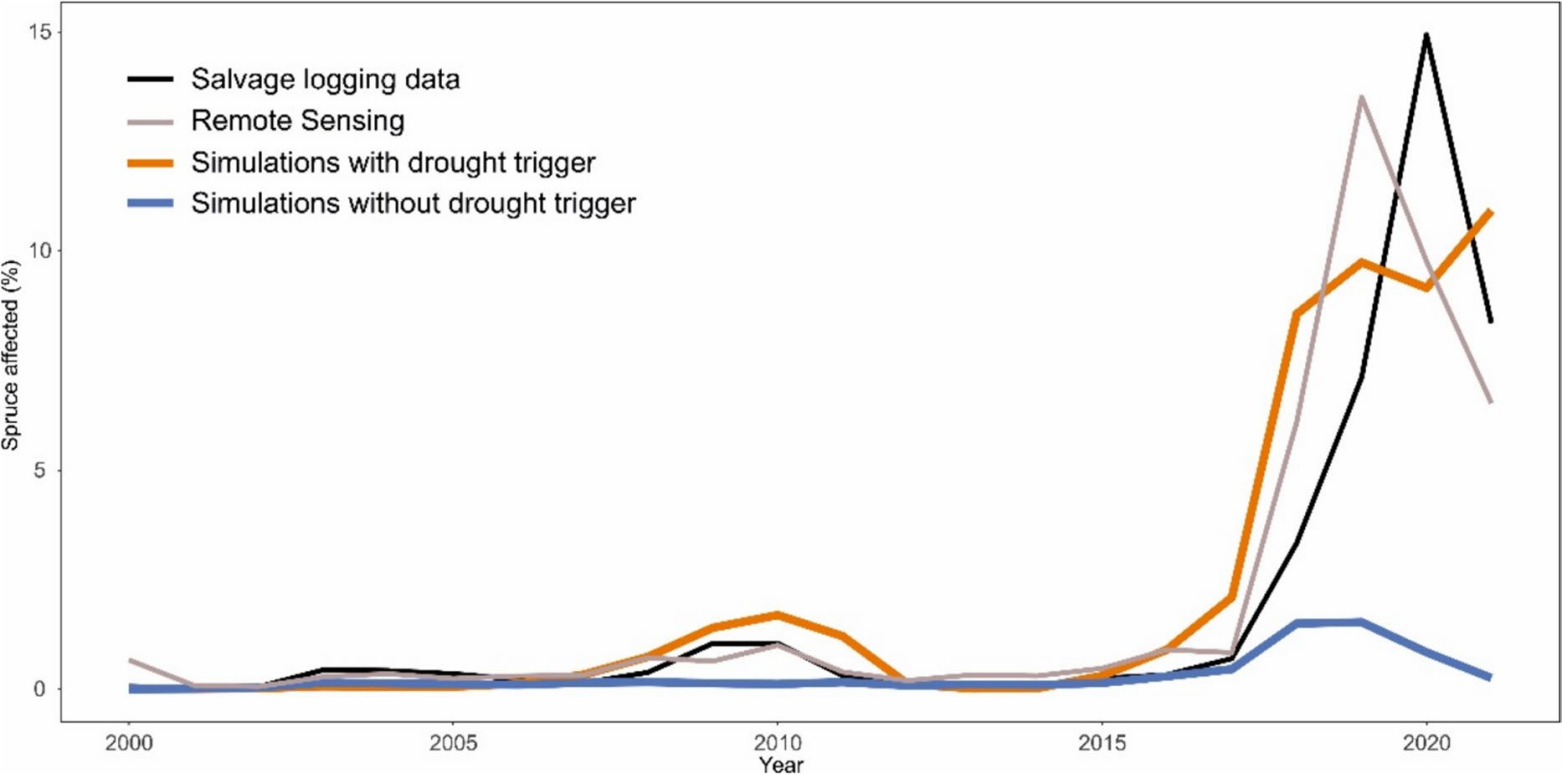
Comparison of simulated bark beetle disturbance data with observed disturbance data. Two simulation outcomes are presented: one considering only wind-incited outbreaks (original model implementation, blue), and another considering both wind- and drought-related outbreaks (orange). The observed disturbance data are based on remote sensing (forest area affected) and field assessments (growing stock affected by bark beetles and salvage logged) in the study area. The annual percentage of spruce growing stock (from simulations and ground data) and of spruce area (from remote sensing data) affected by bark beetles is presented. The simulations were driven by observed climate data from the study landscape

The function parameters were estimated based on an analysis of observed bark beetle disturbance data from our study region (see Fig. 2 and details below), specifically: (i) the annual timber volume of trees killed by bark beetles and salvaged during the period 2000–2021, and (ii) forest area affected by bark beetles identified through the classification of remote sensing data for the period 2000–2021 (Washaya et al. 2024) (Fig. 3). By iteratively comparing simulations to observed data we determined a *VPDjj* of 0.84 kPa to result in the best model performance (parameter *b* in Eq. 1). As the slope parameter *a* had only minor effects on simulated outbreak dynamics (see sensitivity analysis in Online appendix E), we arbitrarily set it to -80 (Fig. 1). Finally, we set *k* to 438, which, after multiplying with the baseline infestation probability of 0.000685, yields the value of 0.3. This represents the upper limit of infestation probability, indicating that drought-stressed trees were equally susceptible to infestation as windfelled trees. As the data required to parameterize distribution functions of the parameters of Eq. 1 are currently lacking, we demonstrated the effect of parameter uncertainty by using the earlier described parameter values as μ_k,_ μ_a,_ and μ_b_, and defining the σ_k_, σ_a_, and σ_b_ as 10% of the respective μ value.

To understand how individual parameters affect simulated bark beetle dynamics, we conducted a sensitivity analysis (Online appendix E) evaluating spruce mortality under a broad range of parameter values. Simulations for the sensitivity analysis did not assume any windthrows and were driven by a set of climatic scenarios from 1961–2100 (Table 1). The response variable used was the total spruce growing stock affected by bark beetles during the simulation. We calculated the first derivative of the resulting response functions (i.e., disturbed growing stock over the range of considered parameter values) to evaluate which parameter value produces the greatest effect.

**Table 1:** Projected changes in climate for 2051–2100 according to three climate models driven by two Representative Concentration Pathway scenarios relative to average climatic conditions for the study landscape in period 1961–1990 (Mean temperature (MT): 8.05 °C; Mean annual precipitation sum (AP): 650 mm; Mean annual Vapour Pressure Deficit (VPD): 0,62 kPa)

Climatic Models / (2051–2100) vs. (1961–1990)	MT - RCP4.5 (℃)	﻿MT - RCP8.5 (℃)	AP - RCP4.5 (%)	AP - RCP8.5 (%)	VPD - RCP4.5 (%)	VPD - RCP8.5 (%)
EC-EARTH-RACMO22E-r1 (hot-wet)	+ 2.5	+ 3.8	− 3.7	+ 1.1	+ 23	+ 27
HadGEM2-CCLM (hot-dry)	+ 3.4	+ 5.0	− 7.9	− 6.8	+ 34	+ 53
MPI-CCLM (cool-dry)	+ 2.4	+ 3.7	− 3.1	− 2.4	+ 24	+ 31
Average	+ 2.8	+ 4.2	− 4.9	− 2.7	+ 27	+ 47

The simulated dynamics was tested against the earlier described disturbance data, aiming at the ability of the model to reproduce the forest mortality wave triggered after 2018 (Fig. 2) in terms of its onset, duration and termination as well as the total proportion of spruce growing stock and area affected. Although independent data would be desirable for model testing, they were not available here. We, however, note that only the critical VPD value (parameter *b*) was estimated based on observations, and the remaining metrics analysed in the evaluation are emergent properties of the simulation.

### Climate data and pattern of outbreak-triggering drought events

To simulate the transition from historical climatic conditions (i.e., without the occurrence of outbreak-triggering droughts) to future climates (with a more prominent role of drought) we conducted simulations for the period 1961–2100. Climate data representing the period 1961–2021 (including daily minimum and maximum temperature, and precipitation) were measured at a meteorological station located in the study landscape (Source: Czech Hydrometeorological Institute). The measured data were downscaled to the 100 × 100m grid cells using the MT-CLIM software (Hungerford 1989), applying lapse rate calculated from twelve weather stations in the surrounding of the study region. Vapour pressure deficit was calculated based on temperature and relative humidity data (Murray 1967). Solar radiation was derived using MT-CLIM for each grid cell based on geographical position and topography.

Climate for the period 2022–2100 was extracted from the FORESEE 4.0 database (Kern et al. 2024), which provides bias corrected climate projections for Central Europe based on the CORDEX initiative (Jacob et al. 2014). The daily data from the nearest grid cell (0.1° × 0.1°) were downscaled to each 100 × 100m grid cell. Future climate was represented by the result of 14 climate models driven by two Representative Concentration Pathways, RCP4.5 and RCP8.5 (Moss et al. 2010). To drive the simulations with iLand, we selected three of the original 14 climate models that captured significant proportion of temperature–precipitation conditions projected in the ensemble (Online appendix B). As each model was driven by two RCP forcings, we considered a total of six distinct climatic trajectories (Table 1). To understand the evolution of outbreak triggering conditions across the twenty-first century, we examined the frequency of critical VPD anomalies (i.e., with *VPDjj* exceeding 0.84 kPa) across all 14 climate models. Specifically, we calculated the proportion of above-threshold climate models for each year from 2022 to 2100, which represent the probability of occurrence of outbreak-triggering conditions.

### Study landscape

The study landscape is located in the Czech Republic, Central Europe (Fig. 2). The size of the landscape is 40,928 ha with 45% forest cover (18 500 ha). The elevation range is 240–540 m a.s.l., and the mean annual air temperature and precipitation sum ranges are 7.8–9.2 °C and 604–683 mm, respectively (1961–2018 data). Forests in the study landscape have been intensively managed for timber production, which resulted in a widespread occurrence of Norway spruce, mostly growing outside of its realized niche. Except for Norway spruce (57.7%), the current tree species composition mainly consists of Scots pine (*Pinus sylvestris* L.; 10.7%), European beech (*Fagus sylvatica* L., 7.6%), and oaks (*Quercus spp.;* 8.8%). The dominant management system is shelterwood (i.e., an even-aged silvicultural system that harvests trees in several progressive steps, leading to the establishment of a new cohort of trees under the shelter of the remaining mature trees) with an average rotation length of 120 years.

After 2018, the landscape was affected by a severe outbreak of spruce bark beetle that was initiated by an extended period of drought (Fig. 2) (Washaya et al. 2024). The outbreak affected 40.6% of spruce growing stock between 2018 and 2021, representing the most severe disturbance in the recorded history of the landscape. The recent disturbance dynamics of the study landscape aligns well with the dynamics observed across the entire country (Washaya et al. 2024) and parts of Europe (Patacca et al. 2023).

### Experimental design

Initial forest conditions were defined based on stand-scale data seamlessly covering the landscape (Source: Forest Management Institute, Czech Republic), which were recorded in the field between 2010 and 2014. The data include the average characteristics of forest stands (i.e., polygons with an average size of 1.04 ha), specifically tree species composition, stand age, and basal area (see Dobor et al. 2024, for more details). To evaluate the transient change in forest dynamics from 1961 to 2100, we used these forest conditions as a starting point for simulations. Simulated management closely resembled the practices applied by managers in the landscape over the past 30 years. It involved a shelterwood cutting system with rotation lengths of 80–140 years, depending on site conditions and tree species. Management was implemented through five site-specific STPs (Rammer and Seidl 2015), which included sequences of operations such as planting after harvests and disturbances, a series of thinning operations, and final harvesting. Unlike current management practices, we did not apply any salvage logging of windfelled trees which serve as breeding substrate for bark beetles (Dobor et al. 2020b, a).

To discern the effect on simulated disturbances resulting from the introduction of drought-induced outbreaks, we compared simulated forest dynamics under wind-induced outbreaks (previous model version) with the new regime that contained the combination of wind- and drought-initiated outbreaks. Given the high climatic sensitivity of bark beetle outbreaks and the anticipated increase in drought conditions under climate change, we assessed simulated disturbance dynamics under climate change in scenarios derived from three climate models for two RCP scenarios (Table 1). Because the parameters in Eq. 1 are represented as probability distributions, each simulation was replicated 30 times with randomly drawn parameter values from these distributions. Altogether, the simulation experiment consisted of 186 runs: [(simulation containing wind trigger only) + (simulation containing drought and wind triggers × 30 replicates of the Eq. 1 parameters)] × (3 RCMs × 2 RCPs).

All simulations were driven by an identical wind sequence representing the timing of windstorms, wind speed, wind direction, and windstorm duration (Seidl et al. 2014). The 140-year long sequence (1961–2100) consisted of a number of minor wind events and six more significant storms (Online appendix D). Wind speed and wind duration parameters were iteratively adjusted to reach the average impact of around 0.5% of the growing stock affected annually within the simulation period 1961–2021, which was the observed level of disturbance in the period 1980–2010. The most severe windstorm occurred in the year 2070, serving to assess the effects of previous forest development driven by different climates and outbreak dynamics on future wind vulnerability of forest growing stock. We note that although all simulations were exposed to the same wind sequence, the simulated impact differs between scenarios because it is an emergent property of the interaction of wind and vegetation conditions.

## Results

### Model testing

There was no significant windthrow in the study landscape during the period 2000–2021 (Fig. 3). After 2017, both remote sensing-based forest change maps and salvage logging data indicated a substantial increase in tree mortality associated with bark beetle outbreaks. During the peak period from 2018 to 2021, between 9 and 13% of spruce growing stock (according to salvage logging data) or spruce area (as indicated by remote sensing) was affected annually. Cumulatively, 44 and 46% of the initial spruce growing stock and spruce area, respectively, were affected between 2000 and 2021.

In simulations using the previous model version (wind-triggered outbreaks only), spruce mortality due to bark beetles slightly increased between 2017 and 2020, peaking at 1.8% of spruce growing stock affected by bark beetles annually. However, this increase was not sufficient to initiate an outbreak, and tree mortality declined as soon as the climatic stress was alleviated. The total affected spruce growing stock between 2000 and 2021 was 6.6% in these simulations. In contrast, spruce mortality caused by bark beetles exhibited a sharp increase post-2018 in simulations which involved a drought trigger (new model version), closely aligning with observed mortality patterns (Fig. 3). The total affected spruce growing stock between 2000 and 2021 was 47.5%, which closely corresponds to observations. Despite substantial difference in the total outbreak impact between the two simulations, the correlations of annual values of simulated tree mortality with observed tree mortality were high in both cases: The R^2^ was of 0.72 without drought trigger and 0.86 with drought trigger.

Finally, while the observed outbreak collapsed after a single culmination year, the simulated outbreak culminated over several years. This is likely the result of the specified outbreak duration in iLand (Kautz et al. 2011; Lausch et al. 2013; Seidl et al. 2016), but could also result from active risk management measures that were not considered in the simulation.

### Temporal pattern of future droughts inciting bark beetle outbreaks

**Fig. 4:**
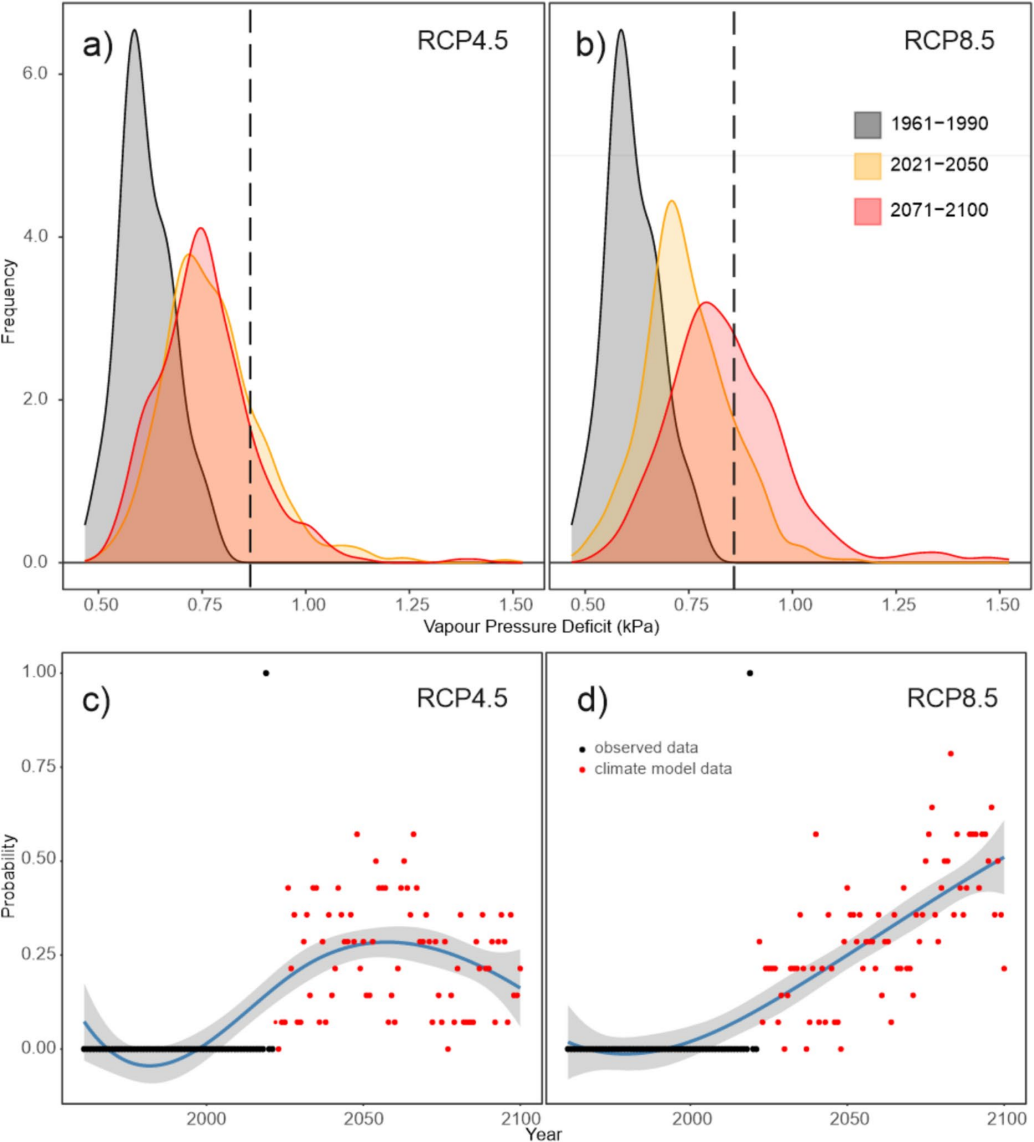
Distribution of two-year weighted mean June-July Vapour Pressure Deficit (VPD_jj_) values across three time periods and two RCP scenarios. The past period 1961–1990 is represented by observed data, while periods 2021–2050 and 2071–2100 are represented by fourteen climate models. Vertical dashed lines indicate the outbreak threshold of 0.84 kPa (**a**, **b**). Panels **c**, **d** give the annual probability of VPDjj values exceeding the outbreak threshold. During 1961–2021, probabilities are binary (0 or 1), while for 2022–2100, they are calculated based on the proportion of climate model runs (n=14) above threshold. A spline function with a 95% confidence band is included to aid the visual interpretation of the data

The distribution of *VPDjj* values differed significantly between the observed climate of the past and climate projections, as well as between RCP scenarios. Under RCP4.5, the median projected *VPDjj* for 2021–2050 (0.77 kPa) was similar to that for 2071–2100 (0.76 kPa) but higher than the observed median of 0.62 kPa for 1961–1990 (Fig. 4a, b). In contrast, under RCP8.5, the median *VPDjj* for 2071–2100 (0.83 kPa) was higher than for 2021–2050 (0.75 kPa). In the period 1961–1990, no year exceeded the outbreak threshold of 0.84 kPa. Under RCP4.5, an average of 24% of years (inter-model range: 7–38%) during 2021–2050 and 19% (0–47%) during 2071–2100 surpassed this threshold. Under RCP8.5, these proportions were 18% (7–27%) and 46% (17–83%), respectively.

Based on agreement across the 14 climate models (Online appendix B), the annual probability of exceeding the outbreak threshold of 0.84 kPa differed between RCP scenarios (Fig. 4c, d). Under RCP4.5, the probability peaked around 2060 at an average of 25%, with some years reaching up to 50%. Under RCP8.5, this proportion steadily increased after 2021, reaching 50% by 2100.

### Simulated disturbance dynamics

**Fig. 5:**
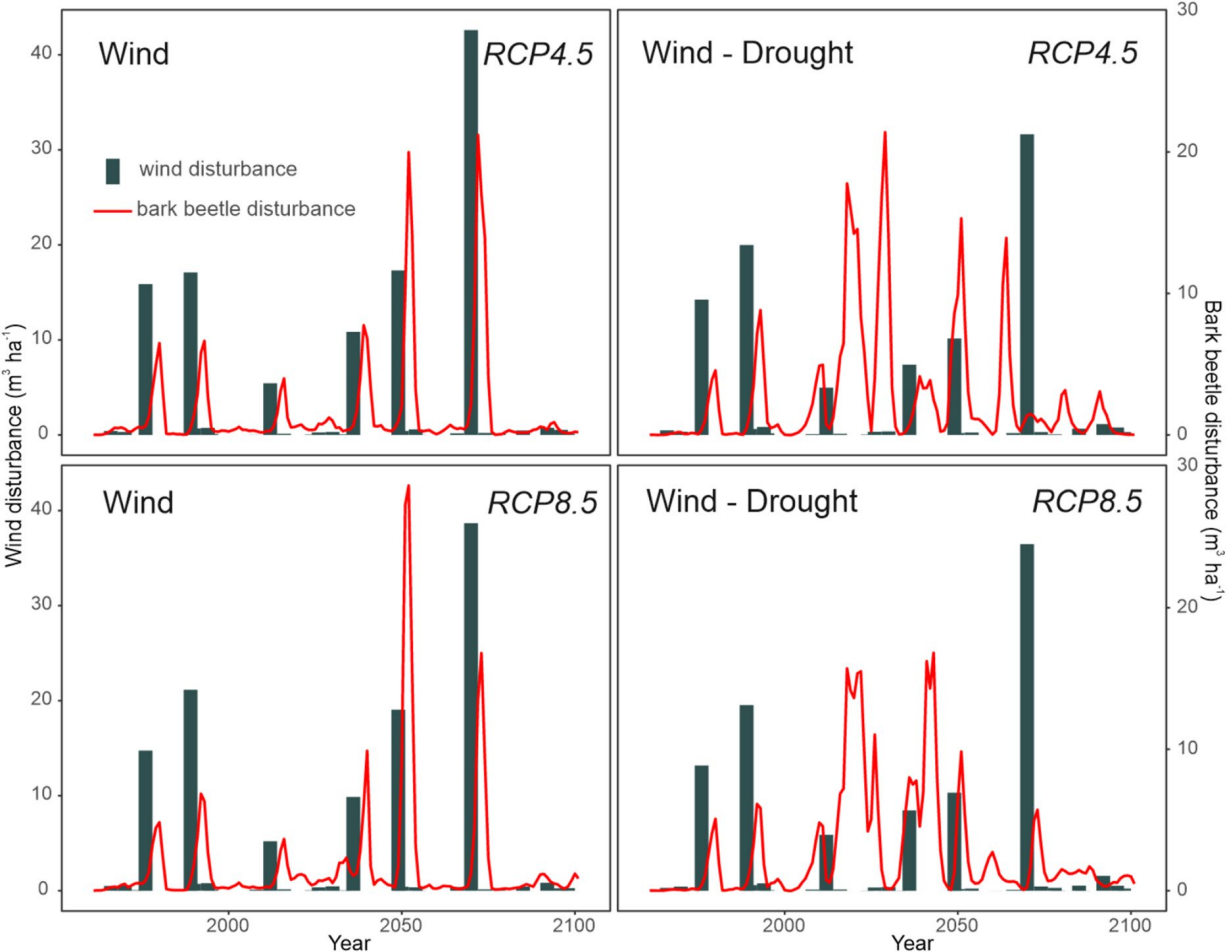
Simulated disturbance dynamics in the period 1961–2100. Two simulations are presented: ‘Wind,’ which includes only wind-initiated bark beetle outbreaks, and ‘Wind-Drought,’ which combines wind and drought as outbreak triggers. In ‘Wind-Drought’, bark beetle activity is increasingly independently from windthrows as triggers. Climate in the period 1961–2021 was defined based on local meteorological data. Climate in the period 2021–2100 was defined based on the climate model HadGEM2_CCLM (hot-dry) driven by the greenhouse gas concentration pathways RCP4.5 and RCP8.5. Simulations driven by other climate model projections can be found in online Online appendix C

**Fig. 6:**
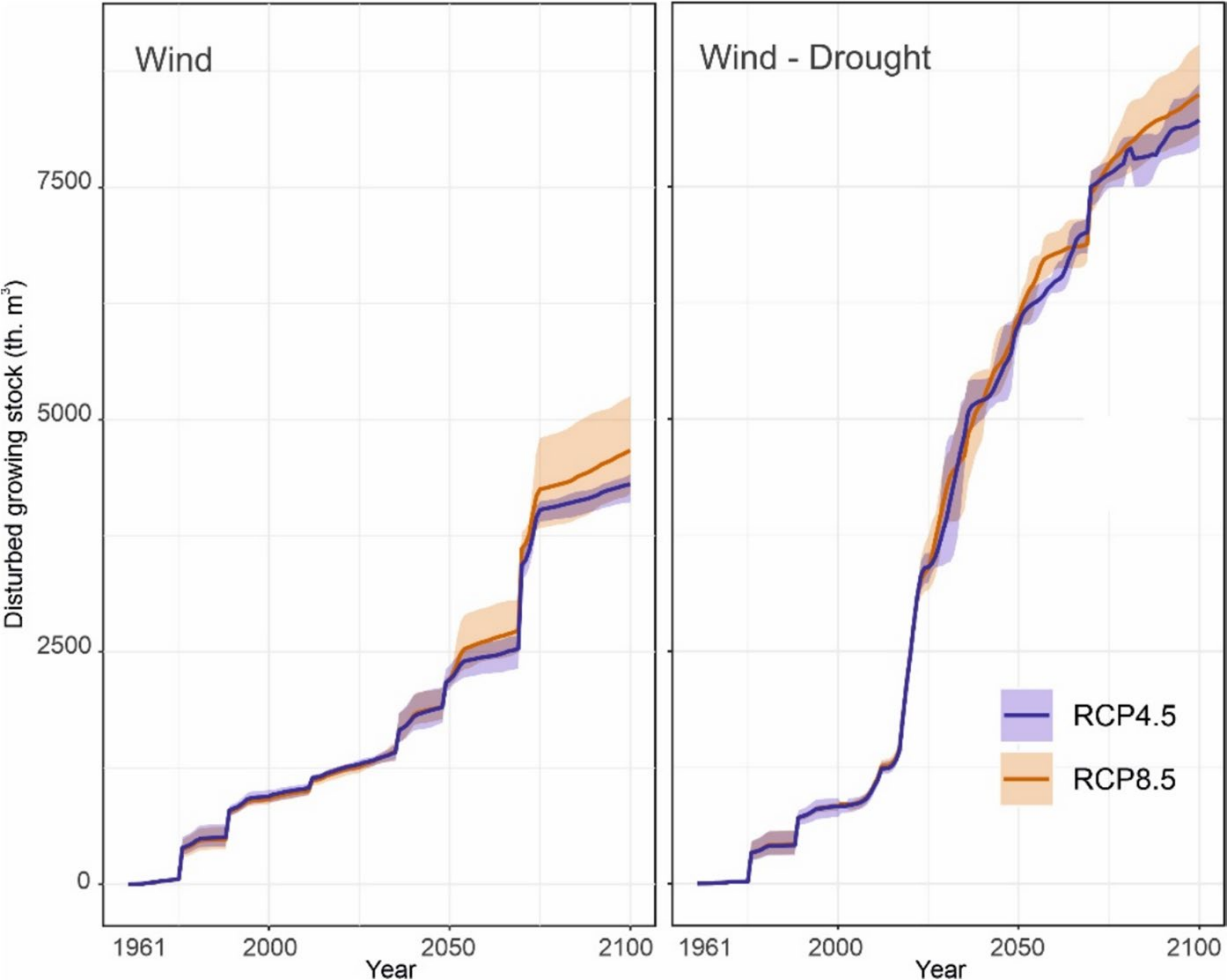
Cumulative disturbed growing stock in the period 1961–2100 under different disturbance and climatic scenarios. ‘Wind,’ includes only wind-initiated bark beetle outbreaks. ‘Wind-Drought’ considers both wind and drought as outbreak triggers. Minimum–maximum envelopes arise from the combination of three climate models nested within RCP4.5 and RCP8.5 climate forcings. In the case of the Wind-Drought scenario, a portion of variability is attributed to the twenty replicates representing parameter uncertainty in the outbreak triggering function (Eq. 1)

In simulations considering only wind as outbreak trigger, bark beetle outbreaks were closely related to windthrows (Fig. 5). Outbreak peaks were higher under RCP8.5 than under RCP4.5 in the second half of the century, when the difference in warming levels between the two RCPs was more pronounced (Online appendix A). The cumulative disturbance impact from 1961 to 2100 ranged between 5,909 and 7,631 thousand m^3^, depending on the climate model (6,323 thousand m^3^, on average) (Fig. 6).

In simulations considering drought as outbreak inciting factor, outbreak size increased, mainly between 2020 and 2060, and outbreaks become distinctly decoupled from wind disturbances. The cumulative disturbance impact exceeded the wind-only scenario by 36.1%, with damage ranging from 8,140.8 to 9,638.8 thousand m^3^. Although outbreak-triggering droughts were more frequent under RCP8.5 than under RCP4.5 (Fig. 4), the difference in outbreak dynamics between the RCP scenarios was only moderate. This is the result of a rapid depletion of host trees for bark beetles even under RCP4.5 (see Sect. “Disturbance effects on growing stock and tree species composition”), constraining disturbance activity across both scenarios, particularly in the second half of the century.

**Fig. 7:**
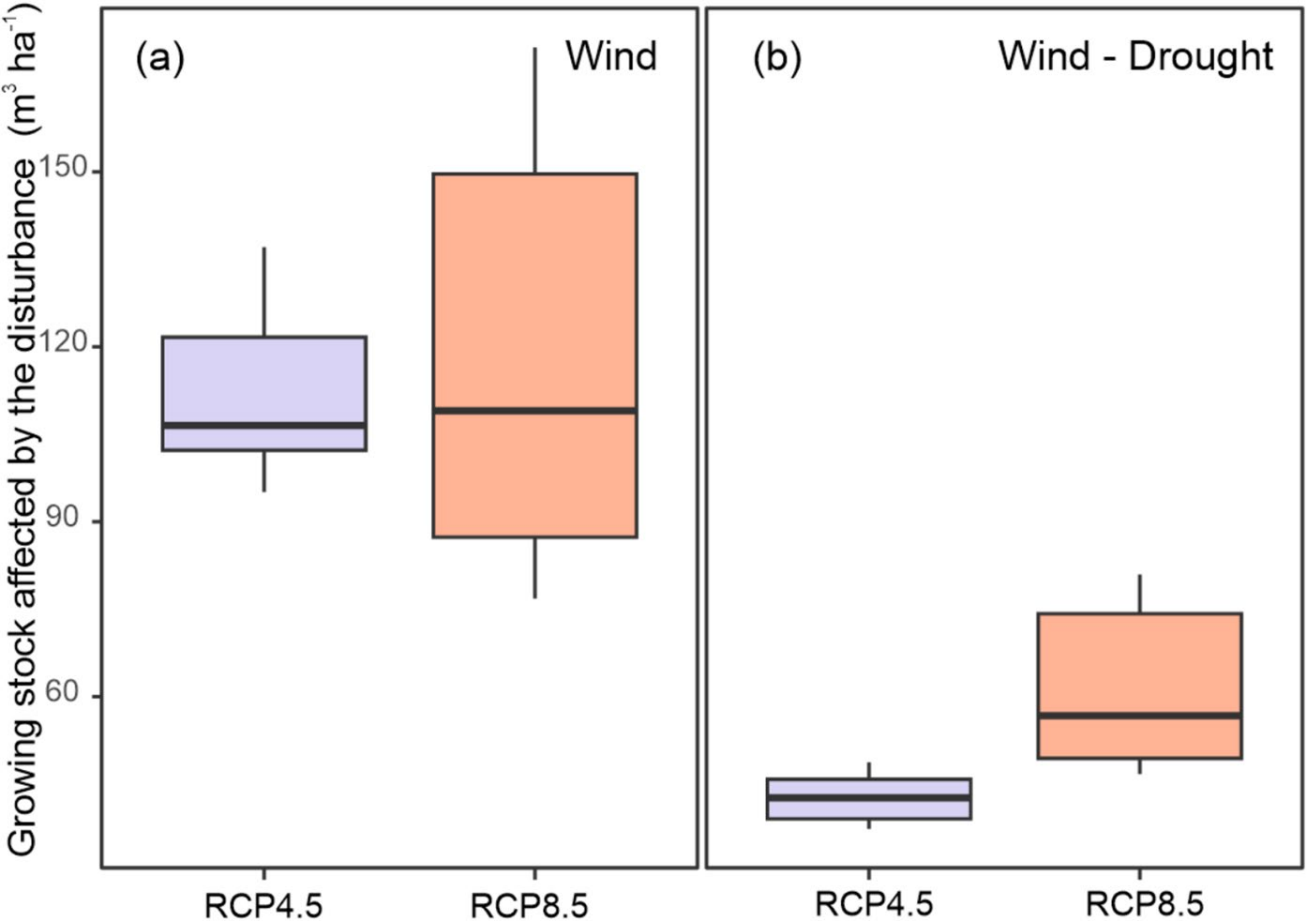
Level of disturbed growing stock during the period 2070–2075 encompassing the major windthrow in year 2070 and the subsequent outbreak. Median, 25–75% and the minimum–maximum ranges are presented. ‘Wind,’ includes only wind-initiated bark beetle outbreaks. ‘Wind-Drought’ combines wind and drought as outbreak triggers. The variation arises from the combination of three climate projections and twenty replicates of each

The combined wind- and drought-induced outbreaks reduced wind impacts in the second half of the twenty-first century (Fig. 5) by depleting wind-prone mature spruce trees, which were replaced by more wind-resistant species (Fig. 9). This suggests a transition from wind- to drought-driven disturbance dynamics in the twenty-first century. This transition was also confirmed by the substantially smaller impact of the major windthrow event simulated in 2070 and the subsequent outbreak (in the years 2070–2075) in simulations considering wind- and drought-induced outbreaks compared to those with wind-induced outbreaks only (Fig. 7).

### Disturbance effects on growing stock and tree species composition

**Fig. 8:**
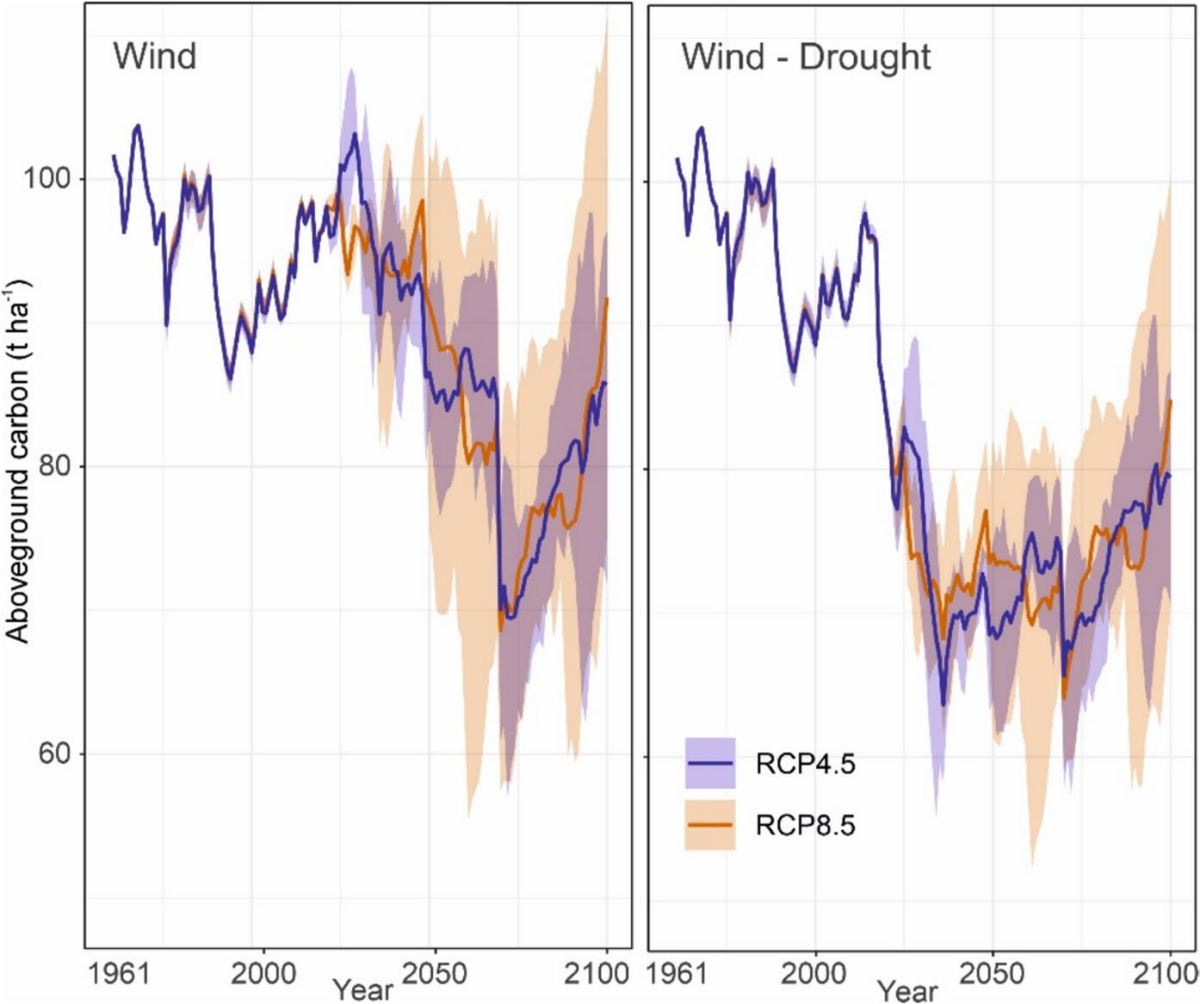
Development of spruce growing stock under different disturbance and climatic scenarios. Mean and minimum–maximum envelopes of simulations driven by three climate models within each RCP scenario (2022–2100) are indicated. ‘Wind’ considers only wind-initiated bark beetle outbreaks. ‘Wind-Drought’ considers both wind and drought as outbreak triggers

While the proportion of Norway spruce was relatively stable between 1961 and 2022, it declined in the remaining period under simulations with both model versions. In the wind-only case, the reduction was significantly accelerated after 2050, when progressive climate change amplified the outbreaks initiated by windthrows (Fig. 8a). The mean spruce reduction by 2100 was 43.1% relative to initial conditions, with an inter-climate model range of 10.2 – 72.1%. This represents a decrease from the initial spruce growing stock of 188 m^3^ ha^−1^ to

**Fig. 9:**
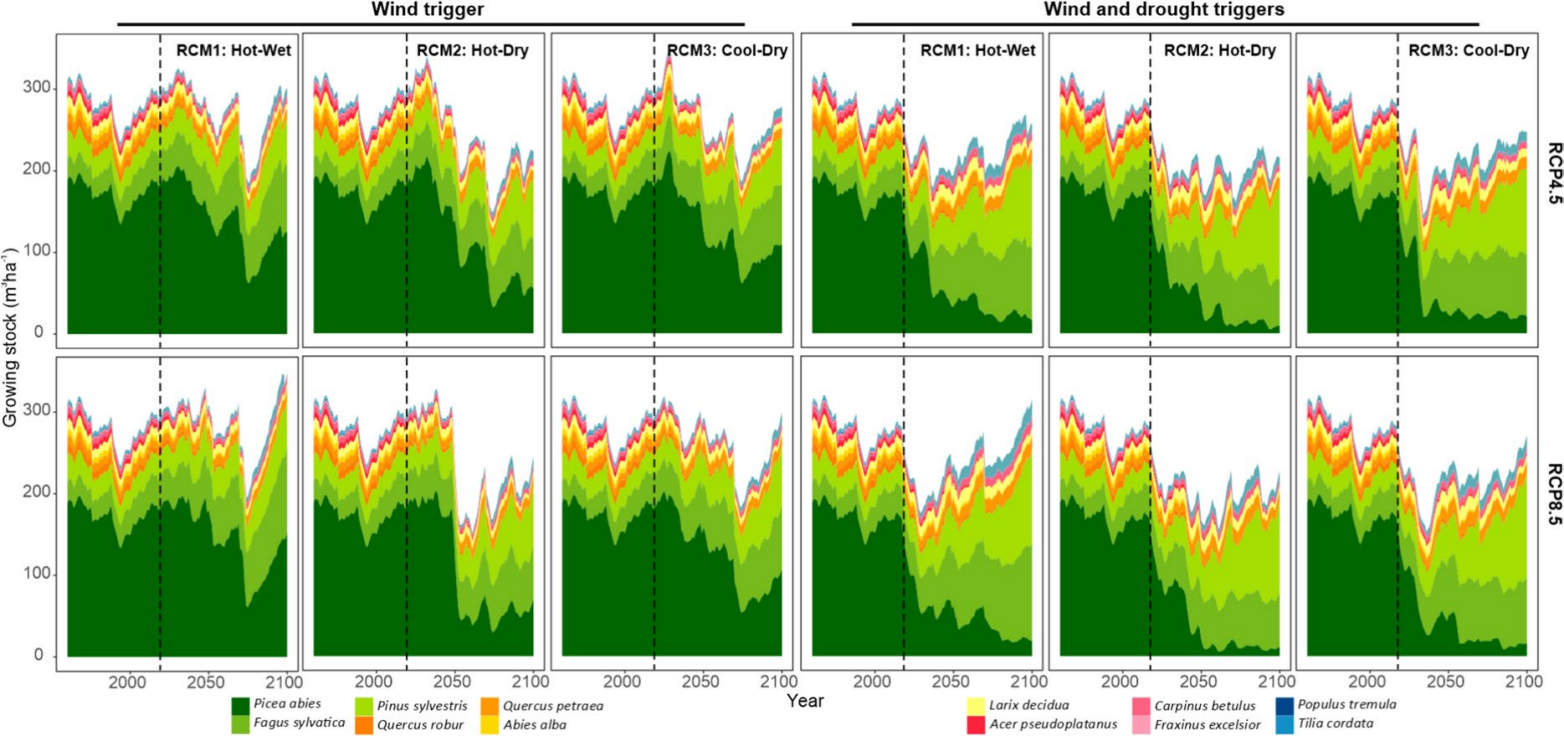
Development of tree species composition simulated with different model versions under different climatic scenarios. ‘Wind,’ considers only wind-initiated bark beetle outbreaks. ‘Wind-Drought’ considers both wind and drought as outbreak triggers. Vertical dashed lines indicate the transition from observed climate data (1961–2021) to climate projections. RCM1: EC_EARTH_RACMO22E_r1, RCM2: HadGEM2_CCLM, RCM3: MPI_CCLM (Table 1)

106.9 m^3^ ha^−1^ (52.4 – 168.8 m^3^ ha^−1^ range). The variation between climate models was substantial. The most extreme scenario, represented by the hot-dry HadGEM2_CCLM model under RCP8.5, led to a sharp reduction in spruce after two consecutive windthrows around 2050, i.e., already before the major windthrow in 2070 (Online appendix C). This reduction persisted until the end of the simulation period. In the less extreme climate scenarios the reduction in spruce was effectively offset by the ingrowth of other tree species, notably European beech and Scots pine, maintaining the overall growing stock at a stable level (Fig. 9). This compensatory dynamic was pronounced under RCP8.5, supported by the stronger fertilization effect of elevated atmospheric CO₂ concentrations.

Simulations also considering drought-incited bark beetle outbreaks (Fig. 8, 9) caused an abrupt reduction of spruce already between 2018–2027, when the first outbreaks triggered by drought emerged. By 2100, 91.7% (range of 88.8—95.5%) of the initial spruce growing stock was lost. Despite the high climate sensitivity of bark beetle disturbances and higher frequency of VPD events above the outbreak threshold under RCP8.5 (Fig. 4), there was no significant difference between the RCP scenarios (Fig. 8b). This can be attributed to the already severe disturbance conditions under RCP4.5, which led to rapid spruce depletion and thus negative feedbacks on disturbance activity. In these simulations, the landscape was transformed into the beech-pine ecosystem, with admixed spruce, larch, and oaks (Fig. 9).

## Discussion

Here, we developed a parsimonious approach that enables the simulation of transitions from wind- to drought-driven bark beetle outbreak dynamics within the forest landscape model iLand (Rammer et al. 2024). The proposed approach can be used to explore, for example, the compounding effects of outbreaks initiated by wind and drought, an interaction expected to become increasingly frequent in the future. We demonstrated that the improved bark beetle module can reliably reproduce observed local outbreak dynamics and generate plausible outcomes in decadal-scale simulations. Given the projected increase in drought conditions under climate change, our approach represents an important contribution to more robust simulations of future disturbance dynamics. In the following, we discuss the limitations of the proposed framework and the potential ecological implications of the presented simulation outcomes.

### Simulation of bark beetle outbreaks

Like several other ecosystem models simulating spruce bark beetle dynamics (Temperli et al. 2013; Maroschek et al. 2015), iLand includes windthrows as an inciting factor for outbreaks. Drought, on the other hand, has typically acted as amplifying factor for bark beetle infestations, modulating disturbance severity rather than triggering large-scale, self-sustaining outbreaks. However, the recent surge in drought-initiated bark beetle outbreaks across Europe underscored the emerging role of severe drought as a primary trigger. In response, we developed and tested a modelling approach capable of initiating outbreaks following such extreme drought events. We chose VPD as an outbreak trigger due to the growing evidence for its significant effects on tree physiology and mortality (Park Williams et al. 2013; Hartmann et al. 2018). Additionally, VPD is employed in iLand to modulate stomatal conductance and primary production, which ultimately determine the availability of non-structural carbohydrate reserves and hence tree defence against bark beetles (Rammer et al. 2024). Although inciting factors, as defined by Manion (1981), operate on a short time scale, such as windthrows, we utilized a two-year weighted average of June-July VPD as outbreak trigger in the simulations. This allowed us to capture the effect of multi-year droughts, which can impair tree physiological processes, including production of secondary metabolites essential for tree defence (Gely et al. 2020). Nonetheless, we note that the degree to which biotic mortality agents are linked to stress physiology is highly variable and insufficiently understood (Trugman et al. 2021), limiting the implementation of these interactions in ecosystem models. For example, legacy effects of previous drought exposure, gradual depletion of non-structural carbohydrate reserves under multiyear droughts, and the relationships between primary and secondary metabolism and their responses to drought, remain insufficiently understood, yet they are essential for improving the modelling of tree–bark beetle interactions (Netherer et al. 2015; Huang et al. 2020). Advances in empirical understanding of these interactions are essential for replacing prevalent, simplified representations (such as the one presented here) with more sophisticated, process-based approaches (Huang et al. 2020). Unless such knowledge is available, the proposed approach, which substitutes complex process understanding with an empirical relationship between changing climate and outbreak risk, provides a practical means to simulate transient disturbance dynamics, with potential applicability beyond bark beetle outbreaks.

We fitted parameter values of the function translating VPD to infestation probabilities, achieving a good match between observed and simulated outbreak dynamics. We note, however, that this parameterization mainly focused on high local accuracy, and that its ability to generalize should be tested in future works. The fact that the dynamics observed for our study landscape aligns well with developments observed across the Czech Republic (Washaya et al. 2024) and Central Europe (Senf et al. 2020) could be a first indication for broader applicability of the approach presented here. Future work should focus especially on refining the VPD outbreak threshold, identified as the most influential parameter in our sensitivity analysis (Online appendix E). To account for uncertainty in outbreak-triggering conditions due to factors such as variable forest age structure, spruce proportion and distribution, and site conditions, we represented each parameter using probability distributions. Due to limited empirical data, these distributions were defined arbitrarily in the current study. However, recent advancements in the use of remote sensing for research on biotic disturbances in forests (Senf et al. 2017) could help identify drought-initiated bark beetle outbreaks across Europe and thus improve the generality of model parameterizations, including parameter uncertainty ranges.

Uncertainty remains regarding the duration of drought-driven bark beetle outbreaks, which may differ from that of wind-driven outbreaks due to different resource distributions (Kärvemo et al. 2023). Moreover, our understanding of the underlying processes causing outbreaks to subside remains insufficient, even for wind-initiated outbreaks (Biedermann et al. 2019). While the outbreak observed in our study landscape peaked in a single year (2019), the simulated tree mortality level remained high until the end of the simulation in 2021. An improved process-based understanding of processes driving the collapse of bark beetle outbreaks could further improve the robustness of simulated trajectories of bark beetle disturbance. In this context, future research should specifically examine how drought- and wind-triggered outbreaks differ in terms of the mechanisms that dampen outbreak dynamics (Biedermann et al. 2019; Schroeder et al. 2025).

Despite the limitations of the proposed framework, our results demonstrate the applicability of iLand and the improved bark beetle module to simulate local developments with high accuracy, and make meaningful decadal-scale projections of landscape trajectories. Although the changes made to the model are parsimonious, they are embedded in a complex simulation framework that incorporates climate-sensitive bark beetle development and tree defence, community level vegetation feedbacks, and possible interactions with other disturbance agents (Honkaniemi et al. 2021), and is thus able to simulate dynamic feedbacks between climate, vegetation and disturbances.

### Effect of drought-initiated outbreaks on forest dynamics

The consideration of drought-induced bark beetle outbreaks significantly amplified simulated bark beetle disturbances after 2018, as the critical VPD threshold was increasingly exceeded. Considering wind- and drought-triggered outbreaks jointly resulted in a persistent reduction of growing stock already in the coming decades. This suggests that while growing stock was resilient to a wind-dominated disturbance regime, this resilience diminished under the compounded impact of wind- and drought-triggered disturbances, even under the moderate climate change represented by RCP4.5. This result aligns with previous studies which indicated low resilience of forest carbon and biomass to novel disturbance regimes under climate change (Turner and Seidl 2023), with potentially strong consequences for bioeconomy and climate change mitigation (Dye et al. 2024; Hetemäki et al. 2024). The simulated magnitude of bark beetle impact also corresponds well to recent observations from Central Europe: For instance, spruce growing stock in the Czech Republic declined by approximately 20% from 2017 to 2022, with certain regions experiencing reductions of 40 to 60% (Washaya et al. 2024).

Incorporating drought as a trigger for bark beetle outbreaks led to a notable decoupling of wind and bark beetle disturbances. Moreover, we observed an overall transition from wind- to drought-driven dynamics in the twenty-first century, with a notably smaller impact of the 2070 windthrow under the combined effect of wind and drought-initiated outbreaks (Fig. 5 and 7). The underlying mechanisms were complex, involving an increasing frequency of critical VPD conditions while windthrow frequency remained stable (Seneviratne et al. 2021), and vegetation feedback characterized by an increase in species which are less wind-prone than spruce (Wallentin and Nilsson 2014), and which are not suitable hosts for the European spruce bark beetle. These findings confirm the ability of our new module to simulate transient ecosystem dynamics and help understand changes in disturbance regimes in Europe caused by climate change.

Regarding tree species composition, spruce remained dominant when solely considering wind-driven bark beetle outbreaks, except in the most severe scenarios of climate change (Fig. 9). The incorporation of drought-incited outbreaks significantly reduced spruce on the landscape, with the major drop occurring between 2030 and 2040, even under the moderate climate scenario represented by RCP4.5. Subsequently, species such as European beech and Scots pine gained dominance. The direction of this transformation aligns well with previous empirical and modelling studies, which indicated the increase in drought tolerant and warm-adapted tree species under climate change, such as European beech at the expense of Norway spruce (Hanewinkel et al. 2013; Kasper et al. 2022). Nevertheless, the high pace of the transition identified here suggests that previous studies may have underestimated the impact of natural disturbances amplified by climate change and their catalyzing effect on forest transformation (Thom & Seidl 2016).

Finally, the two model variants (i.e., with and without considering drought-induced bark beetle outbreaks) resulted in distinctly different variability in simulation outcomes, driven primarily by differences among climatic scenarios. In the wind-only simulations, variability remained high because a substantial amount of mature spruce stands persisted throughout the simulation period, allowing for varied disturbance dynamics under different climatic conditions. Conversely, under the compounding impacts of wind- and drought-initiated outbreaks, spruce growing stock was rapidly depleted—even under RCP4.5—thereby narrowing the simulated range of variability. Hence, while simulations with outbreaks triggered by wind-only suggest that spruce could be maintained and related management objectives achieved under certain conditions, simulations considering both wind and drought as outbreak triggers invariably predict a critical decline of spruce. Since the compounding impacts of wind and drought-triggered outbreaks are likely to become the new normal in the future, these outcomes warrant critical consideration in management planning in spruce dominated forests of Central Europe.

## Conclusion

Modelling novel disturbance dynamics is among the key challenges for current ecosystem modelling, as it is essential for both projecting future ecosystem trajectories and for robustly informing ecosystem management and forestry policy-making. Here, we developed and tested an approach addressing one of the most distinct examples of such an emerging disturbance regime change in Europe: The transition from wind-driven to drought-driven dynamics of the European spruce bark beetle. We introduced this novel outbreak type into an existing simulation framework, and demonstrated that our approach simulates the compound impact of the historically dominating wind-initiated outbreaks and the emerging drought-initiated outbreaks as well as their feedbacks and interactions. While our study contributed a novel approach to simulate bark beetle outbreaks, it also revealed severe limitations in data and system understanding that limit process-based  bark beetle modelling. Our work highlighted that the structure of process-based models needs to be continuously evaluated and potentially updated as novel process-interactions emerge under climate change.

## Supplementary Information

Below is the link to the electronic supplementary material.

Supplementary file1 (DOCX 907 kb)

## Data Availability

The datasets generated during and/or analysed during the current study are available from the corresponding author at request.

## References

[CR1] Asada R, Hurmekoski E, Hoeben AD et al (2023) Resilient forest-based value chains? Econometric analysis of roundwood prices in five European countries in the era of natural disturbances. For Policy Econ 153:102975

[CR2] Augustynczik ALD, Dobor L, Hlásny T (2021) Controlling landscape-scale bark beetle dynamics: Can we hit the right spot? Landsc Urban Plan 209:104035

[CR3] Baier P, Pennerstorfer J, Schopf A (2007) PHENIPS—a comprehensive phenology model of Ips typographus (L.)(Col., Scolytinae) as a tool for hazard rating of bark beetle infestation. For Ecol Manage 249:171–186

[CR4] Bentz BJ, Jönsson AM, Schroeder M et al (2019) *Ips typographus* and *Dendroctonus ponderosae* models project thermal suitability for intra-and inter-continental establishment in a changing climate. Front for Global Change 2:1

[CR5] Biedermann PHW, Müller J, Grégoire J-C et al (2019) Bark beetle population dynamics in the Anthropocene: challenges and solutions. Trends Ecol Evol 34:914–924 31262532 10.1016/j.tree.2019.06.002

[CR6] Blanco JA, Lo Y-H (2023) Latest trends in modelling forest ecosystems: new approaches or just new methods? Curr for Rep 9:219–229

[CR7] Blennow K, Sallnäs O (2004) WINDA—a system of models for assessing the probability of wind damage to forest stands within a landscape. Ecol Model 175:87–99

[CR8] Buras A, Rammig A, Zang CS (2020) Quantifying impacts of the 2018 drought on European ecosystems in comparison to 2003. Biogeosciences 17:1655–1672

[CR9] Dobor L, Hlásny T, Rammer W et al (2020a) Is salvage logging effectively dampening bark beetle outbreaks and preserving forest carbon stocks? J Appl Ecol 57:67–76

[CR10] Dobor L, Hlásny T, Rammer W et al (2020b) Spatial configuration matters when removing windfelled trees to manage bark beetle disturbances in Central European forest landscapes. J Environ Manage 254:109792 31731030 10.1016/j.jenvman.2019.109792 PMC7612771

[CR11] Dobor L, Baldo M, Bílek L et al (2024) The interacting effect of climate change and herbivory can trigger large-scale transformations of European temperate forests. Glob Chang Biol 30:e17194 38385958 10.1111/gcb.17194

[CR12] Dye AW, Houtman RM, Gao P et al (2024) Carbon, climate, and natural disturbance: a review of mechanisms, challenges, and tools for understanding forest carbon stability in an uncertain future. Carbon Balance Manag 19:1–25 39388012 10.1186/s13021-024-00282-0 PMC11468384

[CR13] Gardiner B, Peltola H, Kellomäki S (2000) Comparison of two models for predicting the critical wind speeds required to damage coniferous trees. Ecol Modell 129:1–23

[CR14] Gely C, Laurance SGW, Stork NE (2020) How do herbivorous insects respond to drought stress in trees? Biol Rev 95:434–448 31750622 10.1111/brv.12571

[CR15] Hanewinkel M, Cullmann DA, Schelhaas M-J et al (2013) Climate change may cause severe loss in the economic value of European forest land. Nat Clim Chang 3:203–207

[CR16] Hartmann H, Moura CF, Anderegg WRL et al (2018) Research frontiers for improving our understanding of drought-induced tree and forest mortality. New Phytol 218:15–28 29488280 10.1111/nph.15048

[CR17] Hetemäki L, D’Amato D, Giurca A, Hurmekoski E (2024) Synergies and trade-offs in the European forest bioeconomy research: State of the art and the way forward. For Policy Econ 163:103204

[CR18] Hlásny T, König L, Krokene P et al (2021) Bark beetle outbreaks in Europe: state of knowledge and ways forward for management. Curr for Rep 7:138–165

[CR19] Holuša J, Lukášová K, Lubojacký J (2012) Comparison of seasonal flight activity of *Ips typographus* and *Ips duplicatus*. Sci Agric Bohem 43:109–115

[CR20] Honkaniemi J, Rammer W, Seidl R (2021) From mycelia to mastodons–a general approach for simulating biotic disturbances in forest ecosystems. Environ Model Softw 138:104977

[CR21] Huang J, Kautz M, Trowbridge AM et al (2020) Tree defence and bark beetles in a drying world: carbon partitioning, functioning and modelling. New Phytol 225:26–36 31494935 10.1111/nph.16173

[CR22] Hungerford RD (1989) MTCLIM: A mountain microclimate simulation model. US Department of Agriculture, Forest Service, Intermountain Research Station

[CR23] Jacob D, Petersen J, Eggert B et al (2014) EURO-CORDEX: new high-resolution climate change projections for European impact research. Reg Environ Change 14:563–578

[CR24] Jakoby O, Lischke H, Wermelinger B (2019) Climate change alters elevational phenology patterns of the European spruce bark beetle (*Ips typographus*). Glob Chang Biol 25:4048–4063 31310430 10.1111/gcb.14766

[CR25] Jönsson AM, Appelberg G, Harding S, Bärring L (2009) Spatio-temporal impact of climate change on the activity and voltinism of the spruce bark beetle, *Ips typographus*. Glob Chang Biol 15:486–499

[CR26] Jorgensen SE, Bendoricchio G (2001) Fundamentals of ecological modelling. Elsevier

[CR27] Kärvemo S, Huo L, Öhrn P et al (2023) Different triggers, different stories: Bark-beetle infestation patterns after storm and drought-induced outbreaks. For Ecol Manage 545:121255

[CR28] Kasper J, Leuschner C, Walentowski H et al (2022) Winners and losers of climate warming: Declining growth in Fagus and Tilia vs. stable growth in three Quercus species in the natural beech–oak forest ecotone (western Romania). For Ecol Manage 506:119892

[CR29] Kautz M, Dworschak K, Gruppe A, Schopf R (2011) Quantifying spatio-temporal dispersion of bark beetle infestations in epidemic and non-epidemic conditions. For Ecol Manage 262:598–608

[CR30] Kautz M, Schopf R, Imron MA (2014) Individual traits as drivers of spatial dispersal and infestation patterns in a host–bark beetle system. Ecol Modell 273:264–276

[CR31] Kern A, Dobor L, Hollós R, et al (2024). Seamlessly combined historical and projected daily meteorological datasets for impact studies in Central Europe: The FORESEE v4. 0 and the FORESEE-HUN v1. 0. Clim Serv. 33:100443

[CR32] Landsberg JJ, Waring RH (1997) A generalised model of forest productivity using simplified concepts of radiation-use efficiency, carbon balance and partitioning. For Ecol Manage 95:209–228

[CR33] Lausch A, Heurich M, Fahse L (2013) Spatio-temporal infestation patterns of Ips typographus (L.) in the Bavarian Forest National Park. Germany Ecol Indic 31:73–81

[CR34] Manion PD (1981) Tree Disease Concepts

[CR35] Maroschek M, Rammer W, Lexer MJ (2015) Using a novel assessment framework to evaluate protective functions and timber production in Austrian mountain forests under climate change. Reg Environ Change 15:1543–1555

[CR36] Mezei P, Jakuš R, Pennerstorfer J et al (2017) Storms, temperature maxima and the Eurasian spruce bark beetle *Ips typographus*—An infernal trio in Norway spruce forests of the Central European High Tatra Mountains. Agric Meteorol 242:85–95

[CR37] Moss RH, Edmonds JA, Hibbard KA et al (2010) The next generation of scenarios for climate change research and assessment. Nature 463:747–756 20148028 10.1038/nature08823

[CR38] Murray FW (1967) On the computation of saturation vapor pressure. J Appl Meteorol Climatol 6:203–204

[CR39] Netherer S, Nopp-Mayr U (2005) Predisposition assessment systems (PAS) as supportive tools in forest management—rating of site and stand-related hazards of bark beetle infestation in the High Tatra Mountains as an example for system application and verification. For Ecol Manage 207:99–107

[CR40] Netherer S, Kandasamy D, Jirosová A et al (2004) (2021) Interactions among Norway spruce, the bark beetle Ips typographus and its fungal symbionts in times of drought. J Pest Sci 94:591–614 10.1007/s10340-021-01341-y PMC8550215 34720785

[CR41] Netherer S, Matthews B, Katzensteiner K et al (2015) Do water-limiting conditions predispose N orway spruce to bark beetle attack? New Phytol 205:1128–1141 25417785 10.1111/nph.13166 PMC4315866

[CR42] Netherer S, Lehmanski L, Bachlehner A et al (2024) Drought increases Norway spruce susceptibility to the Eurasian spruce bark beetle and its associated fungi. New Phytol 242:1000–1017 38433329 10.1111/nph.19635

[CR43] Park Williams A, Allen CD, Macalady AK et al (2013) Temperature as a potent driver of regional forest drought stress and tree mortality. Nat Clim Chang 3:292–297

[CR44] Patacca M, Lindner M, Lucas-Borja ME et al (2023) Significant increase in natural disturbance impacts on European forests since 1950. Glob Chang Biol 29:1359–1376 36504289 10.1111/gcb.16531 PMC10107665

[CR45] Peltier DMP, Carbone MS, McIntire CD et al (2023) Carbon starvation following a decade of experimental drought consumes old reserves in Pinus edulis. New Phytol 240:92–104 37430467 10.1111/nph.19119

[CR46] Raffa KF, Aukema BH, Bentz BJ et al (2008) Cross-scale drivers of natural disturbances prone to anthropogenic amplification: the dynamics of bark beetle eruptions. Bioscience 58:501–517

[CR47] Raffa KF, Andersson MN, Schlyter F (2016) Host selection by bark beetles: playing the odds in a high-stakes game. In: Advances in insect physiology. Elsevier, pp 1–74. 10.1016/bs.aiip.2016.02.001

[CR48] Rammer W, Seidl R (2015) Coupling human and natural systems: Simulating adaptive management agents in dynamically changing forest landscapes. Glob Environ Chang 35:475–485

[CR49] Rammer W, Thom D, Baumann M et al (2024) The individual-based forest landscape and disturbance model iLand: Overview, progress, and outlook. Ecol Modell. 10.1016/j.ecolmodel.2024.110785

[CR50] Schroeder M, Knape J, Kärvemo S (2025) Rise and fall of a spruce bark beetle outbreak–Importance of colonisation density and reproductive success. For Ecol Manage 586:122695

[CR51] Schuldt B, Buras A, Arend M et al (2020) A first assessment of the impact of the extreme 2018 summer drought on Central European forests. Basic Appl Ecol 45:86–103

[CR52] Schumann K, Schuldt B, Fischer M et al (2024) Xylem safety in relation to the stringency of plant water potential regulation of European beech, Norway spruce, and Douglas-fir trees during severe drought. Trees 38:607–623

[CR53] Seidl R, Rammer W (2017) Climate change amplifies the interactions between wind and bark beetle disturbances in forest landscapes. Landsc Ecol 32:1485–1498 28684889 10.1007/s10980-016-0396-4 PMC5494037

[CR54] Seidl R, Rammer W, Scheller RM, Spies TA (2012) An individual-based process model to simulate landscape-scale forest ecosystem dynamics. Ecol Modell 231:87–100

[CR55] Seidl R, Rammer W, Blennow K (2014) Simulating wind disturbance impacts on forest landscapes: tree-level heterogeneity matters. Environ Model Softw 51:1–11

[CR56] Seidl R, Müller J, Hothorn T et al (2016) Small beetle, large-scale drivers: How regional and landscape factors affect outbreaks of the European spruce bark beetle. J Appl Ecol 53:530–540 10.1111/1365-2664.12540 PMC4816203 27041769

[CR57] Seneviratne SI, Zhang X, Adnan M, et al (2021) Weather and climate extreme events in a changing climate. 10.1017/9781009157896.013

[CR58] Senf C, Seidl R (2021) Persistent impacts of the 2018 drought on forest disturbance regimes in Europe. Biogeosciences 18:5223–5230

[CR59] Senf C, Seidl R, Hostert P (2017) Remote sensing of forest insect disturbances: Current state and future directions. Int J Appl Earth Obs Geoinf 60:49–60 28860949 10.1016/j.jag.2017.04.004 PMC5572637

[CR60] Senf C, Buras A, Zang CS et al (2020) Excess forest mortality is consistently linked to drought across Europe. Nat Commun 11:6200 33273460 10.1038/s41467-020-19924-1 PMC7713373

[CR61] Sommerfeld A, Rammer W, Heurich M et al (2021) Do bark beetle outbreaks amplify or dampen future bark beetle disturbances in Central Europe? J Ecol 109:737–749 33664526 10.1111/1365-2745.13502 PMC7894307

[CR62] Temperli C, Bugmann H, Elkin C (2013) Cross-scale interactions among bark beetles, climate change, and wind disturbances: A landscape modeling approach. Ecol Monogr 83:383–402

[CR63] Thom D, Seidl R (2016) Natural disturbance impacts on ecosystem services and biodiversity in temperate and boreal forests. Biol Rev 91:760–781 26010526 10.1111/brv.12193 PMC4898621

[CR64] Thom D, Seidl R, Steyrer G et al (2013) Slow and fast drivers of the natural disturbance regime in Central European forest ecosystems. For Ecol Manage 307:293–302

[CR66] Trugman AT, Anderegg LDL, Anderegg WRL et al (2021) Why is tree drought mortality so hard to predict? Trends Ecol Evol 36:520–532 33674131 10.1016/j.tree.2021.02.001

[CR67] Turner MG, Seidl R (2023) Novel disturbance regimes and ecological responses. Annu Rev Ecol Evol Syst 54:63–83

[CR68] Vicente-Serrano SM, Gouveia C, Camarero JJ et al (2013) Response of vegetation to drought time-scales across global land biomes. Proc Natl Acad Sci 110:52–57 23248309 10.1073/pnas.1207068110 PMC3538253

[CR69] Wallentin C, Nilsson U (2014) Storm and snow damage in a Norway spruce thinning experiment in southern Sweden. Forestry 87:229–238

[CR70] Washaya P, Modlinger R, Tyšer D, Hlásny T (2024) Patterns and impacts of an unprecedented outbreak of bark beetles in Central Europe: A glimpse into the future? For Ecosyst 11:100243

[CR71] Wermelinger B (2004) Ecology and management of the spruce bark beetle Ips typographus—a review of recent research. For Ecol Manage 202:67–82

[CR72] Wermelinger B, Seifert M (1999) Temperature-dependent reproduction of the spruce bark beetle Ips typographus, and analysis of the potential population growth. Ecol Entomol 24:103–110

