# Supplementary material for: The increasing role of drought as an inciting factor of bark beetle outbreaks can cause large-scale transformation of Central European forests
Source: Landsc Ecol. 2025 May 23;40(6):108. doi: 10.1007/s10980-025-02125-w (PMC12098194; doi:10.1007/s10980-025-02125-w)
Supplement: Supplementary file 1 — Supplementary file1 (DOCX 907 kb) [file 10980_2025_2125_MOESM1_ESM.docx]

**Appendix A: Driving climate time series**

**
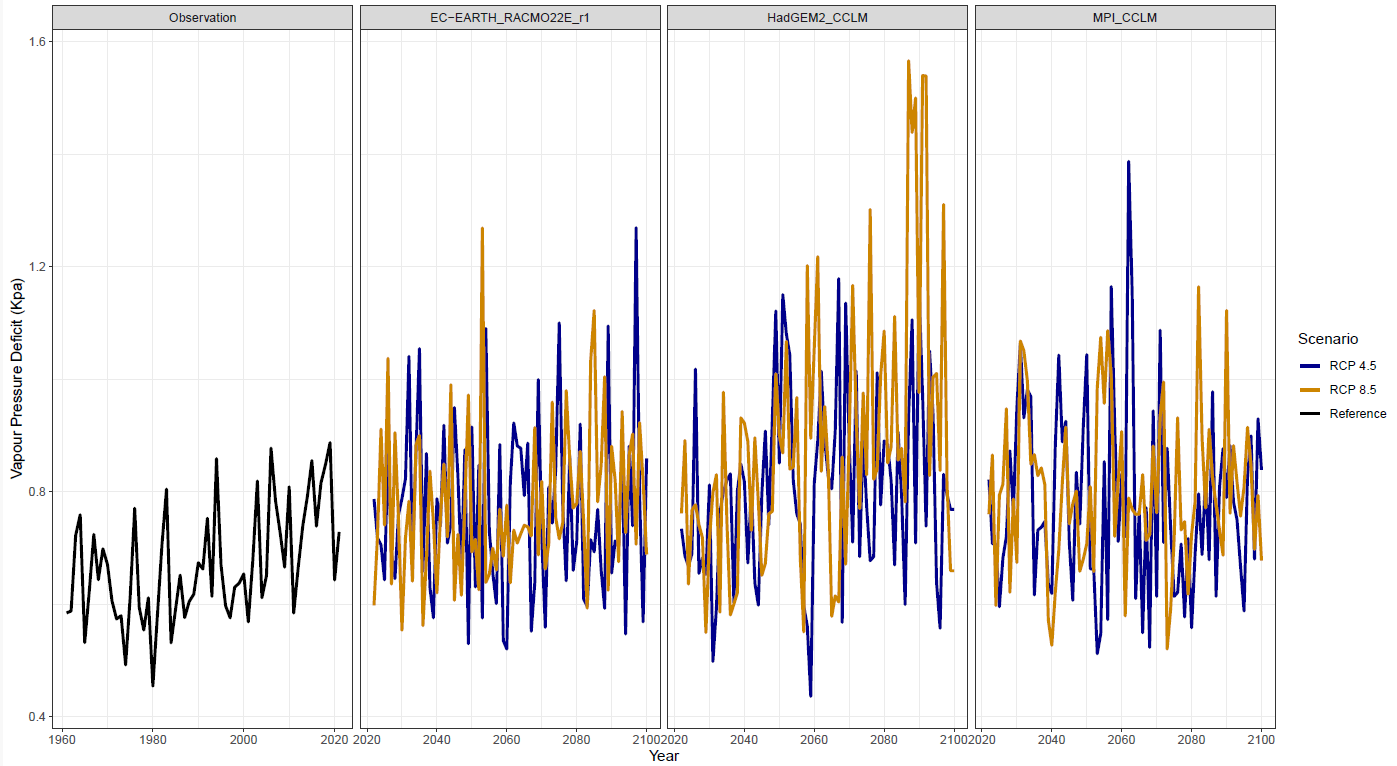
**

**
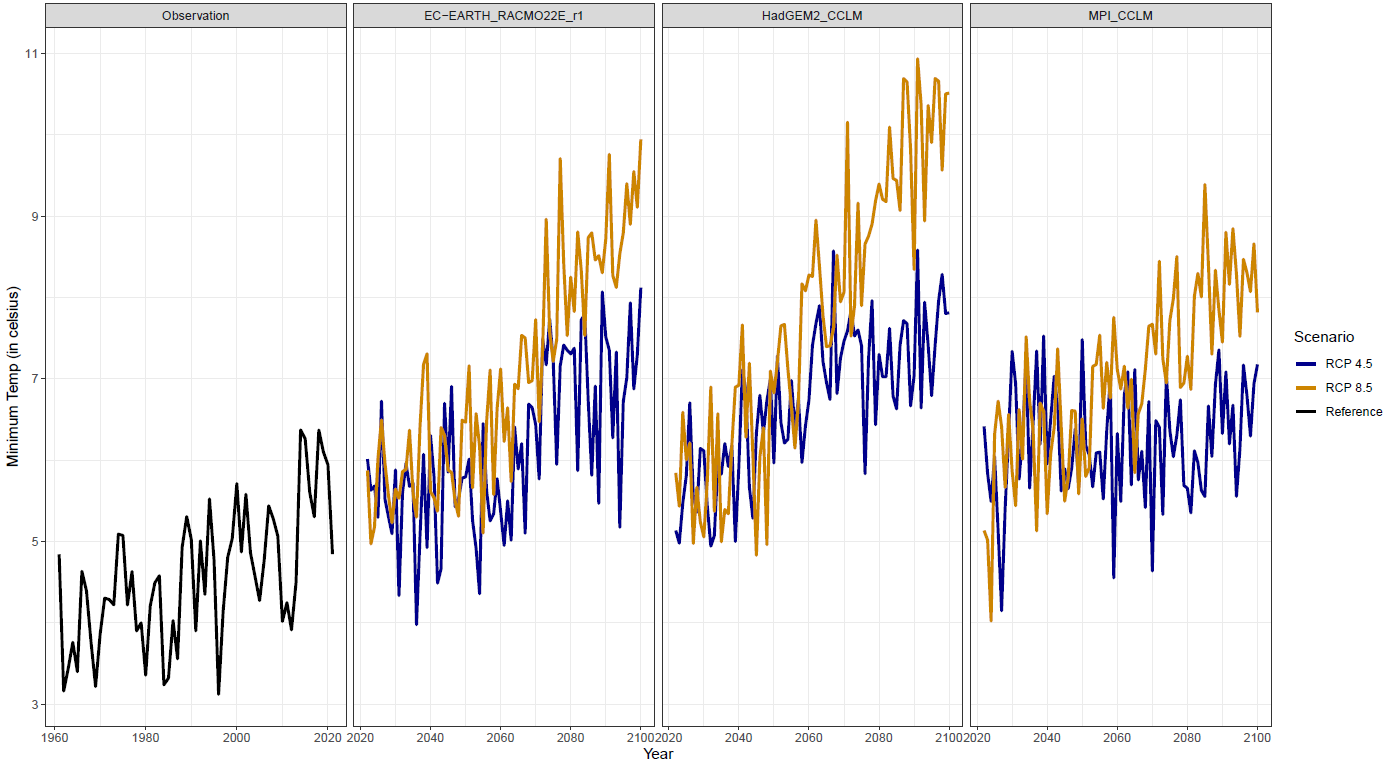
**


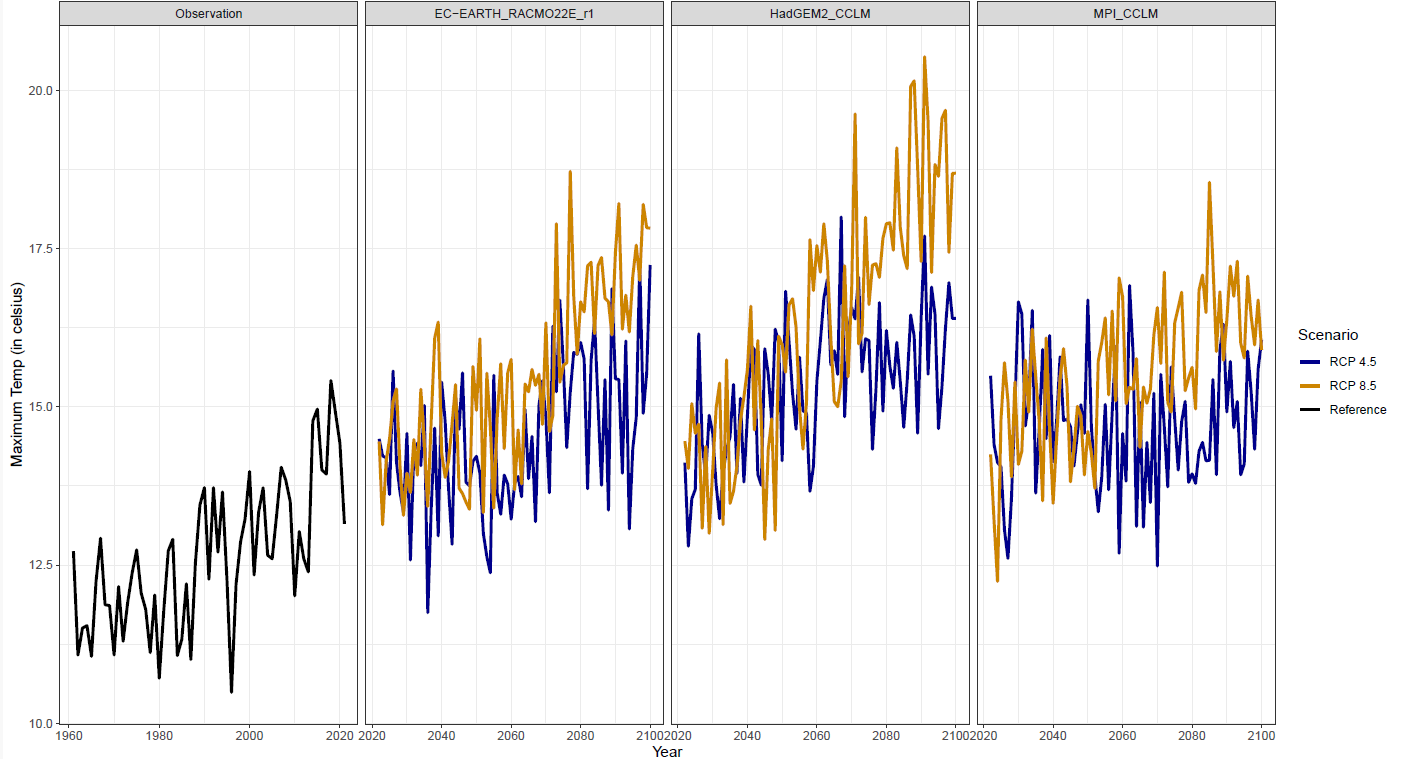


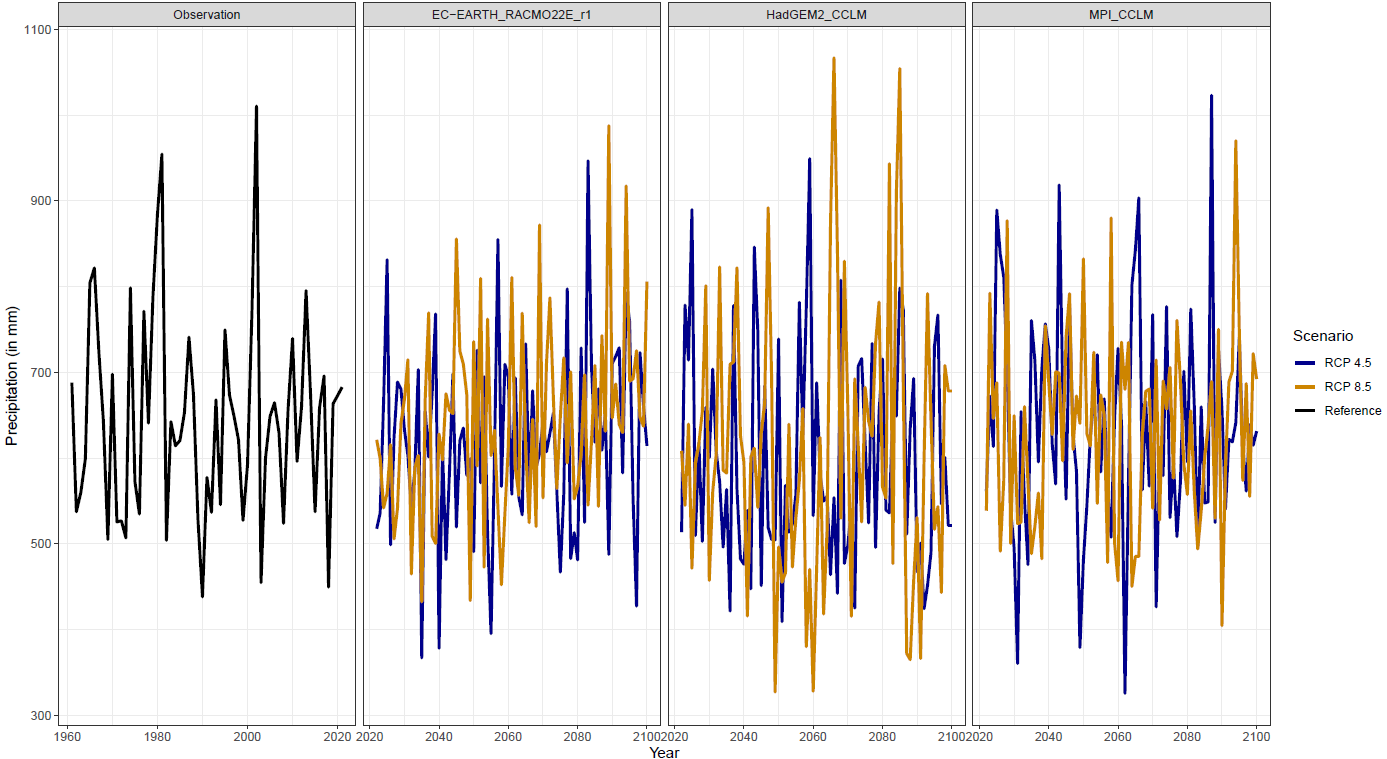


**Fig. A1** Annual time series of vapour pressure deficit, minimum and maximum air temperature, and precipitation for the study landscape. Climate projections (2022-2100) are based on three regional climate models driven by greenhouse gas concentration scenarios RCP4.5 and RCP8.5. The observed data (1961-2021) come from the meteorological station located in the study landscape.

**Appendix B: Temperature-precipitation plot with the position of used climate models**


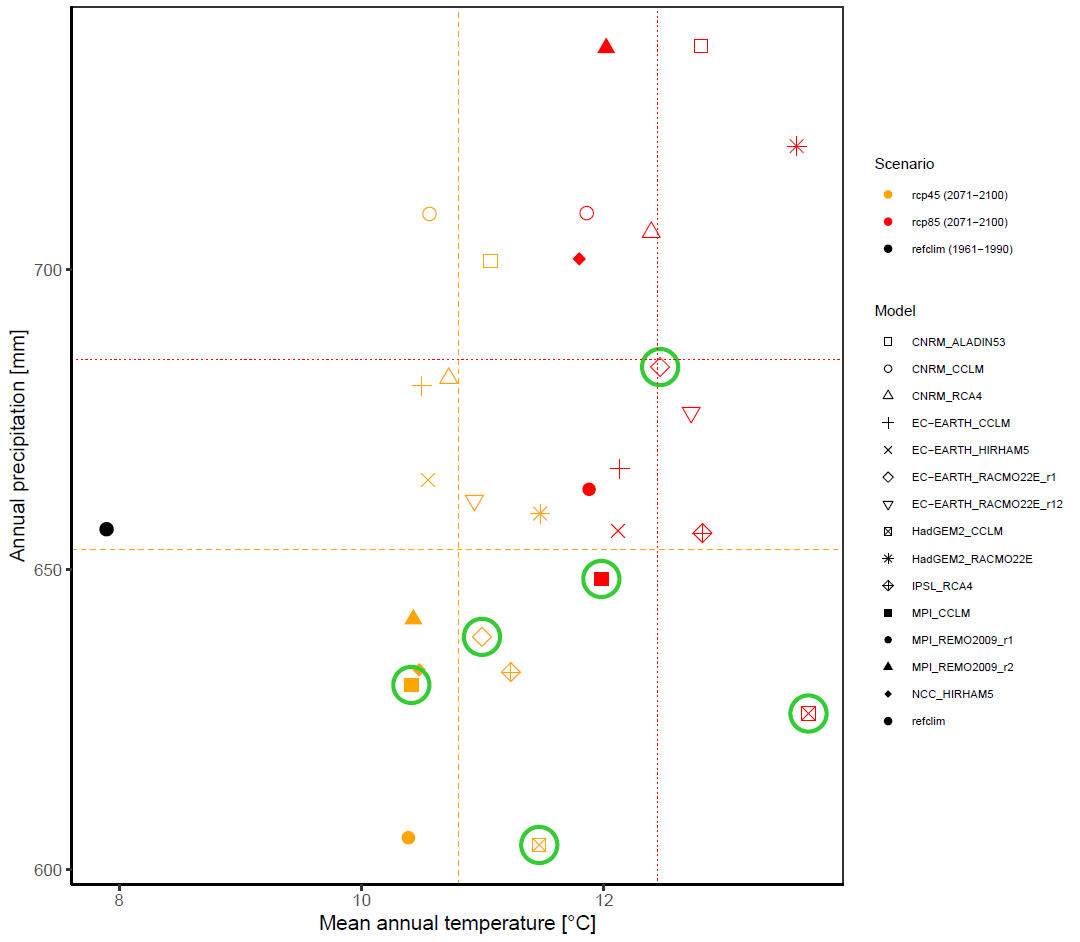


**Fig. B1** Climate models used in the current study. The models highlighted with green circles were used to drive forest development simulations (Table 1 in the main text). All 14 models were used to evaluate the future frequency of bark beetle outbreak triggering conditions based on June-July Vapour Pressure Deficit.

**Appendix C: Simulated disturbance dynamics in the period 1960-2100**


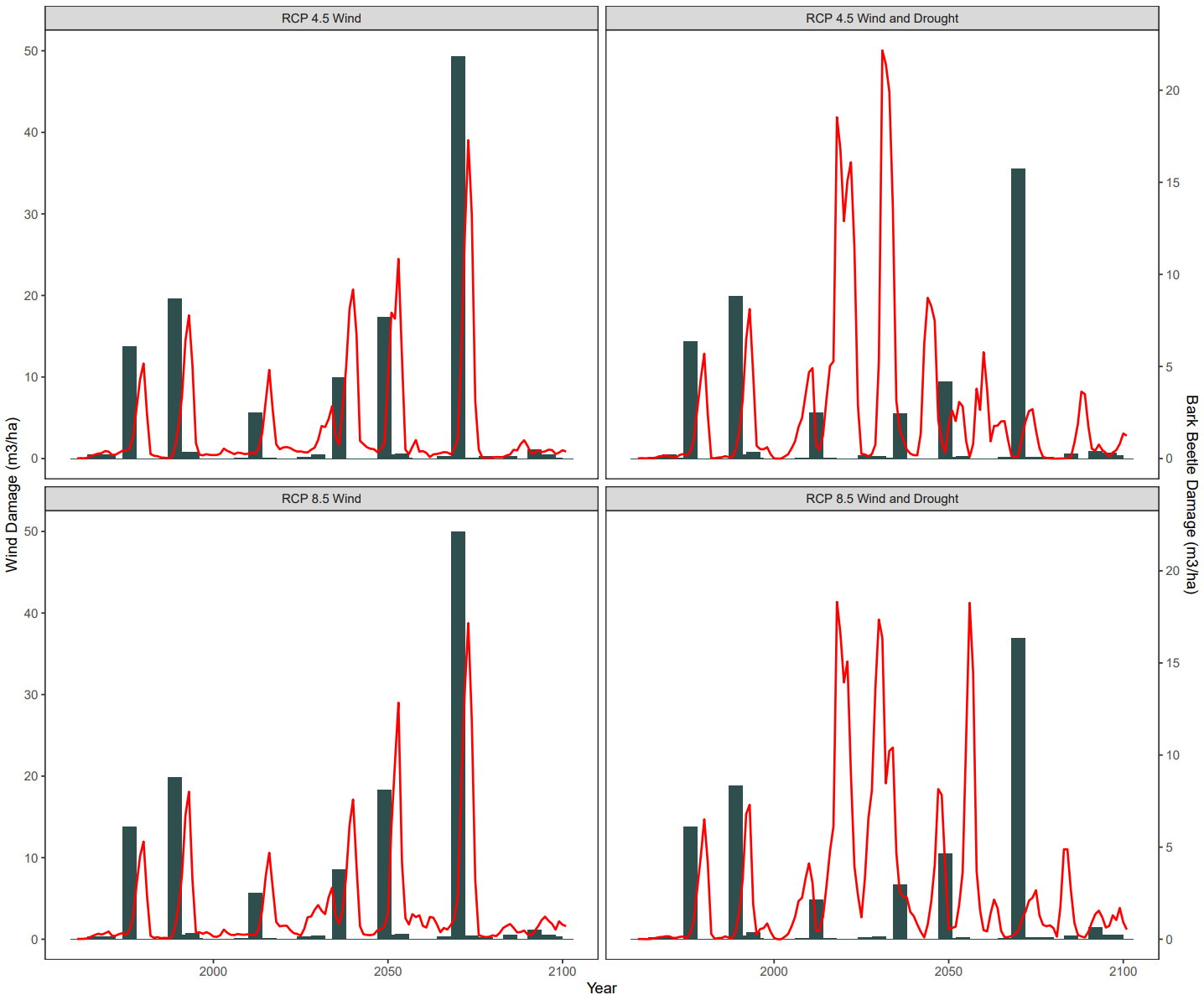


**Fig. C1** Simulated disturbance dynamics in the period 1960-2100. Simulations were driven by climate data produced by climate model MPI-CCLM (see also Fig. 6 in the main text). Black columns represent growing stock affected by wind and red lines represent growing stock affected by bark beetles.


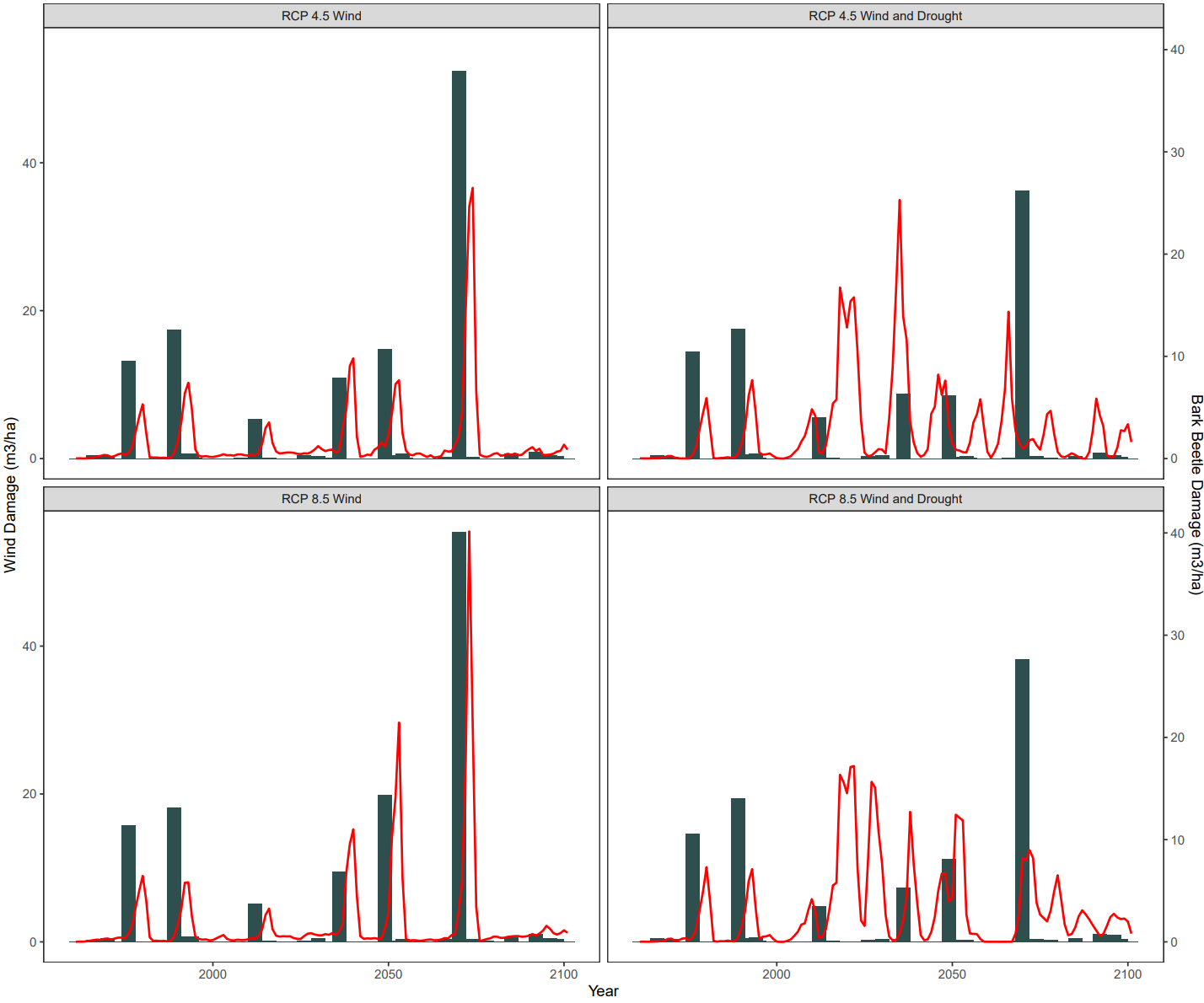


**Fig. C2** Simulated disturbance dynamics in the period 1960-2100. Simulations were driven by climate data produced by climate model EC-EARTH-RACMO22E-r1 (see also Fig. 6 in the main text)

**Appendix D: Parameters of driving wind data**

Table D1 Parameters of driving wind data. The table presents the parameters of wind events distributed across the 140 years long time series (years without wind events were excluded for clarity). The major windthrows are highlighted bold. The remaining events cause only a minor damage.

| **Simulation year** | **Calendar year** | **Day of the year** | **Wind speed (m s^-1^)** | **Wind direction  (°)** | **Wind duration (minutes)** |
| --- | --- | --- | --- | --- | --- |
| 4 | 1964 | 355 | 5.9 | 208.0 | 87.0 |
| 6 | 1966 | 120 | 6.7 | 346.0 | 62.0 |
| 9 | 1969 | 276 | 6.1 | 204.0 | 40.0 |
| 10 | 1970 | 319 | 6.8 | 221.0 | 58.0 |
| 13 | 1973 | 171 | 6.0 | 354.0 | 39.0 |
| 16 | 1976 | 294 | 12.0 | 337.0 | 89.0 |
| 19 | 1979 | 177 | 5.2 | 300.0 | 49.0 |
| 25 | 1985 | 156 | 5.2 | 199.0 | 44.0 |
| **29** | **1989** | **314** | **14.0** | **45.0** | **72.0** |
| 30 | 1990 | 153 | 7.4 | 147.0 | 33.0 |
| 34 | 1994 | 281 | 7.1 | 227.0 | 88.0 |
| 35 | 1995 | 227 | 6.1 | 7.0 | 81.0 |
| 40 | 2000 | 128 | 5.1 | 199.0 | 64.0 |
| 42 | 2002 | 68 | 5.4 | 180.0 | 88.0 |
| 48 | 2008 | 26 | 6.0 | 56.0 | 68.0 |
| 49 | 2009 | 245 | 5.1 | 221.0 | 74.0 |
| 52 | 2012 | 94 | 11.0 | 337.0 | 99.0 |
| 56 | 2016 | 21 | 6.9 | 275.0 | 79.0 |
| 64 | 2024 | 355 | 5.9 | 208.0 | 87.0 |
| 66 | 2026 | 120 | 6.7 | 346.0 | 62.0 |
| 69 | 2029 | 276 | 6.1 | 204.0 | 40.0 |
| 70 | 2030 | 319 | 6.8 | 221.0 | 58.0 |
| 73 | 2033 | 171 | 6.0 | 354.0 | 39.0 |
| **76** | **2036** | **294** | **12.0** | **337.0** | **89.0** |
| 79 | 2039 | 177 | 5.2 | 300.0 | 49.0 |
| 85 | 2045 | 156 | 5.2 | 199.0 | 44.0 |
| **89** | **2049** | **314** | **14.0** | **45.0** | **72.0** |
| 90 | 2050 | 153 | 7.4 | 147.0 | 33.0 |
| 94 | 2054 | 281 | 7.1 | 227.0 | 88.0 |
| 95 | 2055 | 227 | 6.1 | 7.0 | 81.0 |
| 100 | 2060 | 128 | 5.1 | 199.0 | 64.0 |
| 101 | 2061 | 94 | 5.7 | 174.0 | 37.0 |
| 106 | 2066 | 286 | 6.4 | 254.0 | 55.0 |
| **110** | **2070** | **285** | **19.0** | **10.0** | **138.0** |
| 114 | 2074 | 189 | 6.8 | 167.0 | 32.0 |
| 118 | 2078 | 295 | 6.1 | 307.0 | 87.0 |
| 119 | 2079 | 165 | 5.7 | 197.0 | 58.0 |
| 122 | 2082 | 87 | 5.5 | 309.0 | 89.0 |
| 125 | 2085 | 178 | 6.6 | 156.0 | 68.0 |
| 126 | 2086 | 139 | 5.7 | 126.0 | 50.0 |
| 129 | 2089 | 61. | 5.3 | 167.0 | 53.0 |
| 132 | 2092 | 180 | 7.5 | 297.0 | 50.0 |
| 136 | 2096 | 102 | 6.7 | 251.0 | 68.0 |
| 138 | 2098 | 107 | 6.6 | 318.0 | 38.0 |

**Appendix E: Sensitivity analysis, complementary information**

Tab E1 Steps in parameter values used to perform the sensitivity analysis presented in Fig. 4 in the main text. Values that remained constant while the tested parameter was varied are indicated in bold.

| Multiplier | Slope | Inflection Point |
| --- | --- | --- |
| 90 | 20 | 0.34 |
| 160 | 32 | 0.46 |
| 230 | 44 | 0.59 |
| 300 | 56 | 0.71 |
| 370 | 68 | **0.84** |
| **440** | **80** | 0.97 |
| 510 | 92 | 1.09 |
| 580 | 104 | 1.22 |
| 650 | 116 | 1.34 |
| 720 | 128 | 1.47 |

The sensitivity analysis revealed that the parameter *b* of Eq. 1, representing the critical VPD value, had the greatest influence on total tree mortality caused by bark beetles between 2022 and 2100, consistently across both RCP scenarios (Fig. 4). Mortality was highest within the parameter range of 0.3–0.95 kPa, where many years exceeded the critical threshold. Beyond 0.95 kPa, the mortality rate declined sharply, approaching zero at 1.2 kPa. An increase in the slope parameter *a* decreased tree mortality. Increasing the multiplier *k*, which scales the function’s output to determine the final infestation probability, also had a positive effect on mortality. However, the impacts of parameters *a* and *k* were minor compared to the dominant effect of parameter *b*.


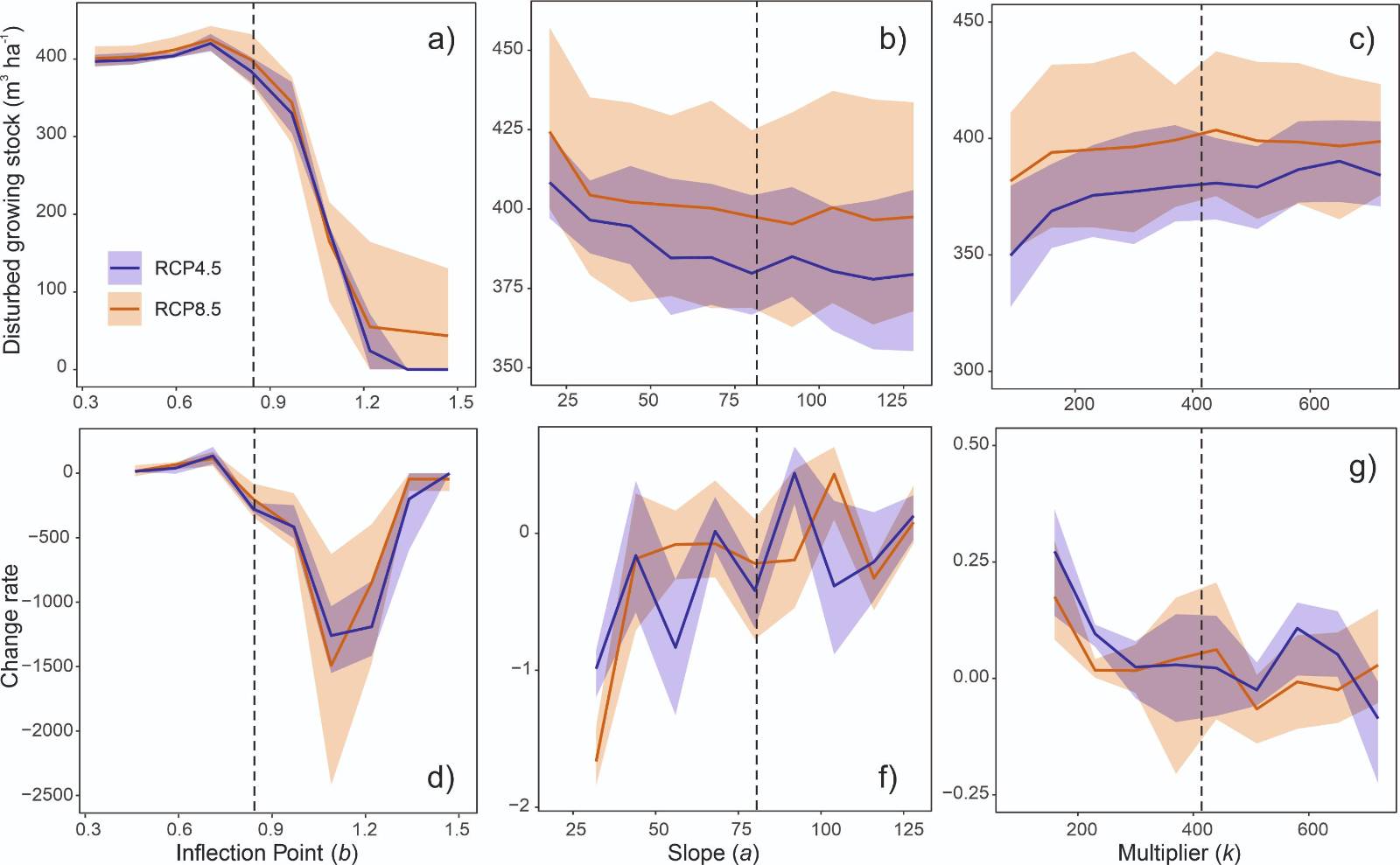


***Fig. E1*** *Response of cumulative tree mortality caused by bark beetles between 2022 and 2100 to variations in parameter values of the function linking Vapour Pressure Deficit to infestation probability (a, b, c). The panels b, c, d indicate the corresponding derivatives of this function, indicating the change rate (negative values indicate damage deceleration, positive values acceleration). Results are presented separately for simulations driven by RCP4.5 and RCP8.5. Shaded areas represent the variability across three Regional Climate Models within each RCP scenario. Vertical dashed lines indicate reference parameter values, which were held constant while varying other parameter values.*
